# Mining and engineering of ene-reductases from marine sediment metagenome for prochiral ACE inhibitor synthesis

**DOI:** 10.1128/aem.02333-25

**Published:** 2026-01-15

**Authors:** Yating Zou, Jinghui Zhou, Yongyi Zeng, Bishuang Chen, Lan Liu, Gang Xu

**Affiliations:** 1 School of Marine Sciences, Sun Yat-Sen University 626303 , Zhuhai, Guangdong, China; 2 Hunan Flag Biotechnology Co., Ltd., Changsha, China; 3 Southern Marine Science and Engineering Guangdong Laboratory - Zhuhai 590852 , Zhuhai, Guangdong, China; University of Milano-Bicocca, Milan, Italy

**Keywords:** marine metagenome, ene-reductase, directed evolution, angiotensin-converting enzyme inhibitor, biotechnology

## Abstract

The development of sustainable biocatalytic processes for pharmaceutical synthesis represents a major goal in green chemistry. Ene-reductases (ERs) are attractive biocatalysts for asymmetric hydrogenation of activated alkenes, yet their industrial application is often constrained by limited substrate scope and stability. In this study, we explored the deep-sea sediment metagenome of the South China Sea and identified 41 putative ER genes, with 22 successfully solubly expressed in *Escherichia coli*. Biochemical characterization revealed broad substrate specificity, achieving up to 90% conversion for diverse α,β-unsaturated compounds. Notably, three enzymes (S2gene2614772, S2gene1139, and S2gene22028) exhibited exceptional adaptability, maintaining high activity over a wide pH range (5.5–8.5) and at low temperatures (15°C). However, none of the wild-type ERs showed significant activity toward the prochiral substrate 2-oxo-4-phenyl-3-butenoic acid, a key intermediate for angiotensin-converting enzyme inhibitors (ACEIs). Through directed evolution, we obtained a mutant (S2gene22028-G102S) with 30-fold enhanced activity, reaching 90% conversion at 10 mM substrate. Scale-up synthesis (5 mmol substrate) afforded 2-oxo-4-phenylbutyric acid (OPBA) at 11 mg/mL, demonstrating industrial potential. This study highlights marine metagenomes as valuable sources of novel ERs and provides an efficient biocatalytic route to ACEI precursors.

**IMPORTANCE:**

The development of sustainable biocatalysts for pharmaceutical synthesis is a pivotal goal in green chemistry. This study leverages the untapped enzymatic diversity of the South China Sea deep-sea sediment metagenome to discover novel ene-reductases (ERs). We not only identified robust ERs with broad substrate promiscuity and exceptional adaptability to low temperature and pH fluctuations but also successfully engineered a variant to overcome the key biocatalytic challenge in the synthesis of 2-oxo-4-phenylbutyric acid (OPBA), a critical precursor to angiotensin-converting enzyme inhibitors. Our work underscores marine metagenomes as a valuable reservoir for discovering industrially relevant biocatalysts and demonstrates the power of combining metagenomic mining with protein engineering to enable greener manufacturing routes for high-value pharmaceuticals.

## INTRODUCTION

The pursuit of efficient and sustainable pharmaceutical synthesis has positioned biocatalysis as a pivotal technology in green chemistry characterized by mild reaction conditions, low environmental impact, and operational ease (1, 2). The ACS Green Chemistry Institute’s Pharmaceutical Roundtable has specifically emphasized selective hydrogenation under mild conditions as a critical research goal, displaying the importance of novel biocatalysts reducing carbon-carbon bonds (3). In particular, ene-reductases (ERs, EC 1.6.99.1) have attracted considerable interest for their ability to catalyze the asymmetric hydrogenation of activated alkenes, a key step in synthesizing valuable industrial intermediates (4, 5). However, the widespread use of ERs is still hindered by inherent limitations, including narrow substrate specificity, insufficient stability, and difficulty in processing sterically demanding molecules common in pharmaceutical contexts (6, 7).

Marine ecosystems, particularly microbial communities in unique environments like the South China Sea, harbor immense enzymatic diversity shaped by extreme physicochemical conditions (e.g., high salinity, low oxygen, and hydrostatic pressure) (8–10). Metagenomic approaches have revolutionized access to this untapped resource, enabling the discovery of enzymes from uncultured microorganisms (11, 12). Recent studies have identified marine-derived oxidoreductases with exceptional properties, such as salt tolerance and cold adaptability, which are highly desirable for industrial processes (13–16). However, ERs from marine sources remain largely unexplored, representing a missed opportunity to expand the biocatalytic toolbox.

**Fig 1:**
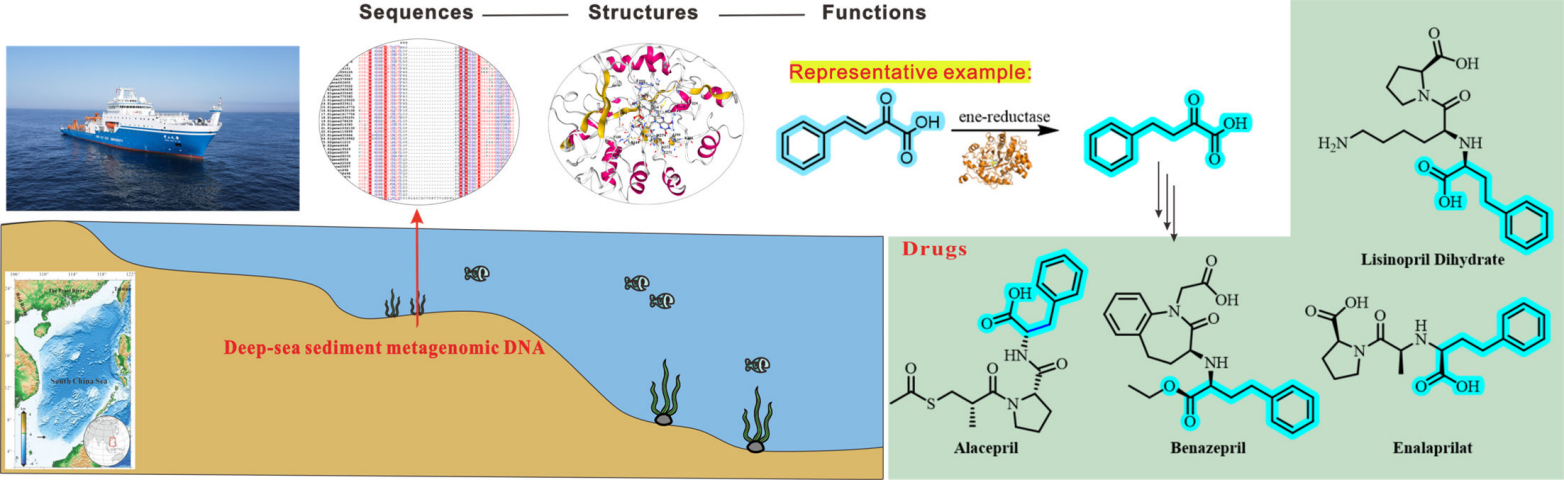
A case study in marine enzyme development: ERs for the hydrogenation of activated alkenes, especially the synthesis of 2-oxo-4-phenylbutyric acid, which serves as a prochiral intermediate in the production of angiotensin-converting enzyme inhibitors (ACEI).

2-Oxo-4-phenylbutyric acid (OPBA), a prochiral β-ketoacid, serves as a pivotal intermediate in the synthesis of angiotensin-converting enzyme inhibitors (ACEI), including blockbuster drugs like enalapril and lisinopril (17–19) (Fig. 1). Conventional chemical synthesis of OPBA derivatives relies on transition metal-catalyzed hydrogenation, which is constrained by inherent limitations, including the high cost of noble metal catalysts and the occurrence of undesirable side reactions involving excessive reduction of the ketone group (20, 21). While ERs are theoretically ideal for this transformation, no known natural ERs efficiently produce OPBA due to steric hindrance from the phenyl moiety. This gap highlights the need for discovering and engineering ERs with tailored activity.

Continuing our long-standing interest in the application of marine enzymes (22–27), and in conjunction with our recent interest in the synthesis of pharmaceutical intermediates (28–30), we engaged in the discovery, engineering, and application of marine-derived ERs in prochiral ACEI intermediate synthesis (Fig. 1). Here, we present the first systematic investigation of marine-derived ERs for OPBA synthesis. By integrating metagenomic mining of South China Sea deep-sea sediment, high-throughput enzyme characterization, and iterative protein engineering, we identified ER variants with high activity toward OPBA. Further process optimization enabled scalable synthesis, demonstrating industrial feasibility. Our work not only establishes a sustainable route to ACEI intermediates but also underscores marine ecosystems as a rich source of novel biocatalysts for pharmaceutical applications. 

## MATERIALS AND METHODS

### Chemicals and reagents

NAD(P)H, 2-oxo-4-phenyl-3-butenoic acid, and 2-oxo-4-phenylbutyric acid (OPBA) were obtained from Aladdin Reagents (Shanghai, China) and Hunan Flag Biotechnology (HNFLAG), respectively. The Phanta Max Master Mix DNA polymerase and *Escherichia coli* BL21(DE3) competent cells were from Vazyme Biotech (Nanjing, China). Oligonucleotide synthesis and DNA sequencing were provided by Tsingke Biological Technology (Beijing, China). All other chemicals and solvents were of analytical grade or higher and sourced from Bide, Aladdin, or Macklin.

### South China Sea sediment metagenomic DNA extraction and sequencing

Deep-sea sediment samples (2,400–4,000 m depth) were collected from the South China Sea during the Open Cruise in June–July 2022 aboard RV Zhong Shan Da Xue. Total genomic DNA was extracted with the Mag-Bind Soil DNA Kit (Omega Bio-tek, USA), and its concentration, purity, and quality were assessed using a TBS-380 fluorometer, NanoDrop2000, and 1% agarose gel electrophoresis, respectively.

The DNA was fragmented to ~350 bp using a Covaris M220 instrument (Gene Company Limited, China) for paired-end library preparation with the NEXTFLEX Rapid DNA-Seq Kit (Bioo Scientific, USA). After adapter ligation, paired-end sequencing was performed on an Illumina NovaSeq platform at Majorbio Bio-Pharm Technology Co., Ltd. (Shanghai, China) using the NovaSeq 6000 S4 Reagent Kit (300 cycles) as per manufacturer guidelines.

### Sequence quality control and genome assembly

Data analysis was performed using the free online Majorbio Cloud Platform (https://www.majorbio.com/). Briefly, adapter sequences and low-quality reads (length < 50 bp or quality value < 20) were trimmed from the paired-end Illumina reads using fastp (version 0.23.0; https://github.com/OpenGene/fastp). Metagenomic assembly was conducted with MEGAHIT (version 1.1.2; https://github.com/voutcn/megahit), which employs succinct de Bruijn graphs. Contigs with a length ≥ 300 bp were retained as the final assembly output and subsequently used for gene prediction and annotation.

### *In silico* metagenomic screening for novel ene-reductases

Open reading frames (ORFs) were predicted from the assembled contigs using Prodigal (https://github.com/hyattpd/Prodigal). Predicted ORFs with a length ≥ 100 bp were extracted and translated into amino acid sequences using EMBOSS 6.6.0 (https://emboss.sourceforge.net/) with the standard National Center for Biotechnology Information (NCBI) genetic code.

The amino acid sequences of three known ene-reductases (OPR1 [NCBI ID: NP_001234781.1], OYE3 [NCBI ID: NP_015154.1], and YqjM [NCBI ID: WP_255003398.1]) were used as query templates for standalone BLASTP searches against the predicted ORFs. The candidate enzyme sequences were selected based on the following criteria: sequence identity to the queries between 10 and 80%, alignment length between 200 and 600 amino acids, query coverage greater than 90%, and an *E*-value cutoff of <1e−10. Subsequently, the conserved domains of the candidate sequences were analyzed using the SMART database to confirm the presence of typical ene-reductase domains. The amino acid sequences of the 41 putative ene-reductases identified in this study are included in the supplemental material (Section 4).

### Phylogenetic analysis

The phylogenetic tree was constructed using the neighbor-joining method in MEGA 11 software (31), with bootstrap support based on 1,000 replicates. The resulting tree was subsequently visualized and annotated using the Interactive Tree of Life (iTOL) platform (32).

### Gene synthesis and expression of the putative 41 ene-reductases

The genes encoding the ERs were codon-optimized for *E. coli*, synthesized, and cloned into the pET28a(+) expression vector by Tsingke Biotechnology Co., Ltd. (Beijing, China). Each gene was inserted between the *NdeI* and *HindIII* restriction sites to generate an in-frame fusion with an *N*-terminal His-tag. Drawing on relevant literature (26, 27), this study employs a two-step cultivation system comprising “LB small-scale cultivation (LB-SC) and TB large-scale cultivation (TB-LC)”: LB-SC screens seed solutions of highly viable, pure strains, while TB-LC facilitates efficient product synthesis via optimizing nutritional conditions and environmental parameters.

Single colonies of the recombinant strains were inoculated into 5 mL of LB medium (33) supplemented with 50 µg/mL kanamycin and cultured at 37°C with shaking at 200 rpm for 5 h. The seed culture was then transferred into 250 mL of TB medium (34) containing 50 µg/mL kanamycin at a 2% (v/v) inoculum size, followed by incubation at 37°C and 200 rpm for 3 h. Gene expression was induced by adding 0.2 mM isopropyl-β-d-thiogalactopyranoside (IPTG), leading to the production of the target protein. And the culture was further incubated at 17°C and 180 rpm for 20 h. Cells were harvested by centrifugation at 4°C and 12,000 × *g* for 10 min. To obtain the expressed recombinant protein, the cell pellet was resuspended in phosphate-buffered saline (PBS; 50 mM, pH 7.5) and disrupted by ultrasonication on ice using a JY92-IIDN Ultrasonic Cell Disruptor. The process was carried out at a power of 75% in pulse mode (3 s on and 7 s off) for a total duration of 30 min to ensure efficient cell lysis while minimizing protein denaturation. Cell debris was removed by centrifugation at 12,000 × *g* for 20 min. The supernatant containing the soluble His-tagged enzyme was applied to a 5 mL Ni-NTA column (Sangon Biotech) pre-equilibrated with loading buffer (20 mM sodium phosphate, 500 mM NaCl, 25 mM imidazole, pH 7.5). The column was washed with washing buffer (20 mM sodium phosphate, 500 mM NaCl, 50 mM imidazole, pH 7.5) to remove non-specifically bound proteins. The target protein was then eluted using elution buffer (20 mM sodium phosphate, 500 mM NaCl, 250 mM imidazole, pH 7.5). The fractions containing the pure protein were combined and subjected to ultrafiltration for desalting, during which the buffer was exchanged with 50 mM PBS (pH 7.5) for at least 2 cycles to thoroughly remove imidazole. The purified protein was stabilized with 20% (w/v) glycerol, aliquoted, and stored at −80°C for subsequent use. The purity and concentration of the protein were analyzed by SDS-PAGE and a Bradford Assay Kit (Quick Start, Bio-Rad, USA), respectively.

### Catalytic activity assay for overexpressed ene-reductases

The specific activity of the ene-reductases was determined by monitoring the consumption of NAD(P)H at 340 nm (*ε* = 6.22 mM⁻¹‧cm⁻¹). The reaction mixture consisted of 1 mL of 50 mM PBS buffer (pH 7.5) containing 2 mM substrate and 0.2 mM NADH. The reaction was initiated by adding 0.01–1 mg of enzyme. For crude enzyme assays, the crude lysate (100 mg wet cells/mL) was centrifuged, and 5–50 µL of the supernatant (corresponding to 1 mg/mL total protein concentration of the crude lysates) was added to the reaction mixture containing 2 mM substrate and 0.2 mM NADH. Enzyme activity was determined by measuring the initial rate of change in absorbance at 340 nm. All assays were conducted at 30°C in triplicate. One unit (U) of enzyme activity was defined as the amount of enzyme required to consume 1 μmol of NAD(P)H per minute.

To account for any background activity from native *E. coli* proteins, control reactions were performed in parallel using crude lysates prepared from *E. coli* BL21(DE3) cells harboring the empty pET28a(+) vector following the same expression and lysis procedure. The specific activities reported for all recombinant ERs represent the net values obtained after subtracting this background activity.

### Optimization of enzymatic reduction reaction conditions

The initial reaction conditions were set as follows: 10 mM substrate (4-phenyl-3-buten-2-one), 50 mM PBS buffer (pH 8.0), 12 mM NADH, reaction temperature of 30°C, and reaction time of 16 h. Reactions were performed in 2-mL centrifuge tubes with a total volume of 1 mL. Based on these conditions, further optimization was carried out to evaluate the effects of pH, temperature, and cofactor.

### General procedure for enzymatic reduction of α,β-unsaturated compounds

The enzymatic reduction was conducted in 50 mM PBS buffer (pH 7.5) containing 10 mM substrate, 1 mg·mL⁻¹ purified ERs, and 12 mM NADH. The reaction mixture was incubated at 30°C with shaking at 700 rpm for 16 h. After the reaction, the mixture was extracted with twice its volume of ethyl acetate and dried over anhydrous Na_₂_SO_₄_. The organic phase was then analyzed by gas chromatography (GC). Alternatively, the mixture was appropriately diluted, filtered through a 0.22 μm membrane, and subjected to high-performance liquid chromatography (HPLC) analysis. Detailed analytical methods are provided in the supplementary information (Fig. S5 through S15).

### Protein structure prediction and molecular docking

The three-dimensional (3D) structures of the ene-reductases were predicted using AlphaFold2. Molecular docking of the substrate 2-oxo-4-phenyl-3-butenoic acid and the cofactor FMN into the enzyme active sites was carried out with AutoDock 4.2 (Scripps Institute, California, USA). Ligand-receptor complexes exhibiting reasonable interaction patterns and relatively low binding energies were selected for further analysis. Structural visualization and analysis were performed using PyMOL (Version 2.0.7).

### Site-directed mutation of S2gene22028 and S1gene1252928

Site-directed mutagenesis was carried out using an overlap extension PCR approach with the primers provided in the supplementary information (Table S1). The PCR was performed in a 25 μL reaction system containing 0.5–1.0 ng of plasmid template, 1 μL of each mutagenic primer (10 μM), 12.5 μL of 2× Phanta Max Master Mix DNA polymerase, and 10.5 μL of H_₂_O. The amplification protocol consisted of an initial denaturation at 95°C for 3 min, followed by 30 cycles of denaturation at 95°C for 15 s, annealing at the Tm (melting temperature of the non-overlapping sequences) for 15 s, and extension at 72°C for 3.5 min, with a final extension at 72°C for 5 min. The resulting plasmid was transformed into *E. coli* BL21(DE3) and verified by sequencing.

### OPBA synthesis using ERs and their mutants

The catalytic activity of the 12 ene-reductases and their mutants toward 2-oxo-4-phenyl-3-butenoic acid was evaluated. Each 1 mL reaction mixture contained 10 mM substrate, 12 mM NADH, and 1 mg purified enzyme in 50 mM PBS buffer (pH 7.5). Reactions were carried out at 30°C with shaking at 700 rpm for 24 h. After appropriate dilution, the mixture was filtered through a 0.22 μm membrane and analyzed by HPLC using an Agilent 1260 Infinity II System equipped with a DAD detector and an Agilent InfinityLab Poroshell 120 phenyl-hexyl column (35°C). The mobile phase consisted of (A) H_₂_O with 0.1% acetic acid and (B) acetonitrile (21% B) run at a flow rate of 0.5 mL/min. Detection was performed at 210 and 280 nm; note that only the substrate exhibits absorption at 280 nm, while OPBA does not.

### Analytical methods

The conversion of substrates and the formation of products were analyzed by GC or HPLC, as specified for each substrate. Detailed chromatographic conditions for each compound (including instrument, column, temperature program, mobile phase, and flow rate) are provided in Section 1 of the supplemental material.

For quantification, authentic standards of both the substrate and the product were commercially sourced and used to establish their respective retention times. The yield of the desired product was calculated by using calibration curves.

## RESULTS AND DISCUSSION

### Mining ene-reductases from an untapped marine resource

Aligning with the goal of discovering sustainable catalysts, we turned to the deep-sea sediment metagenome of the South China Sea, an extreme environment likely to host robust enzymes. This approach avoids the resource-intensive cultivation of microorganisms, representing a greener starting point for catalyst discovery (35).

To identify new ERs, we used three previously characterized enzymes, namely, OPR1 (36), OYE3 (37), and YqjM (38), as query probes targeting highly conserved catalytic (Tyr192) and substrate-binding residues (His187, His190, or Asn190) typical of ene-reductases (39–41). Through sequence-based screening of metagenomic data derived from South China Sea microbial communities, we identified 41 putative ene-reductase genes (see Fig. S1 for sequence alignment). Notably, a BLASTP search against the NCBI non-redundant protein database confirmed that all these putative enzymes are previously uncharacterized, with most sequences exhibiting no significant homology to documented proteins. The complete amino acid sequences of these putative ERs are included in the supplemental material (Section 4).

**Fig 2:**
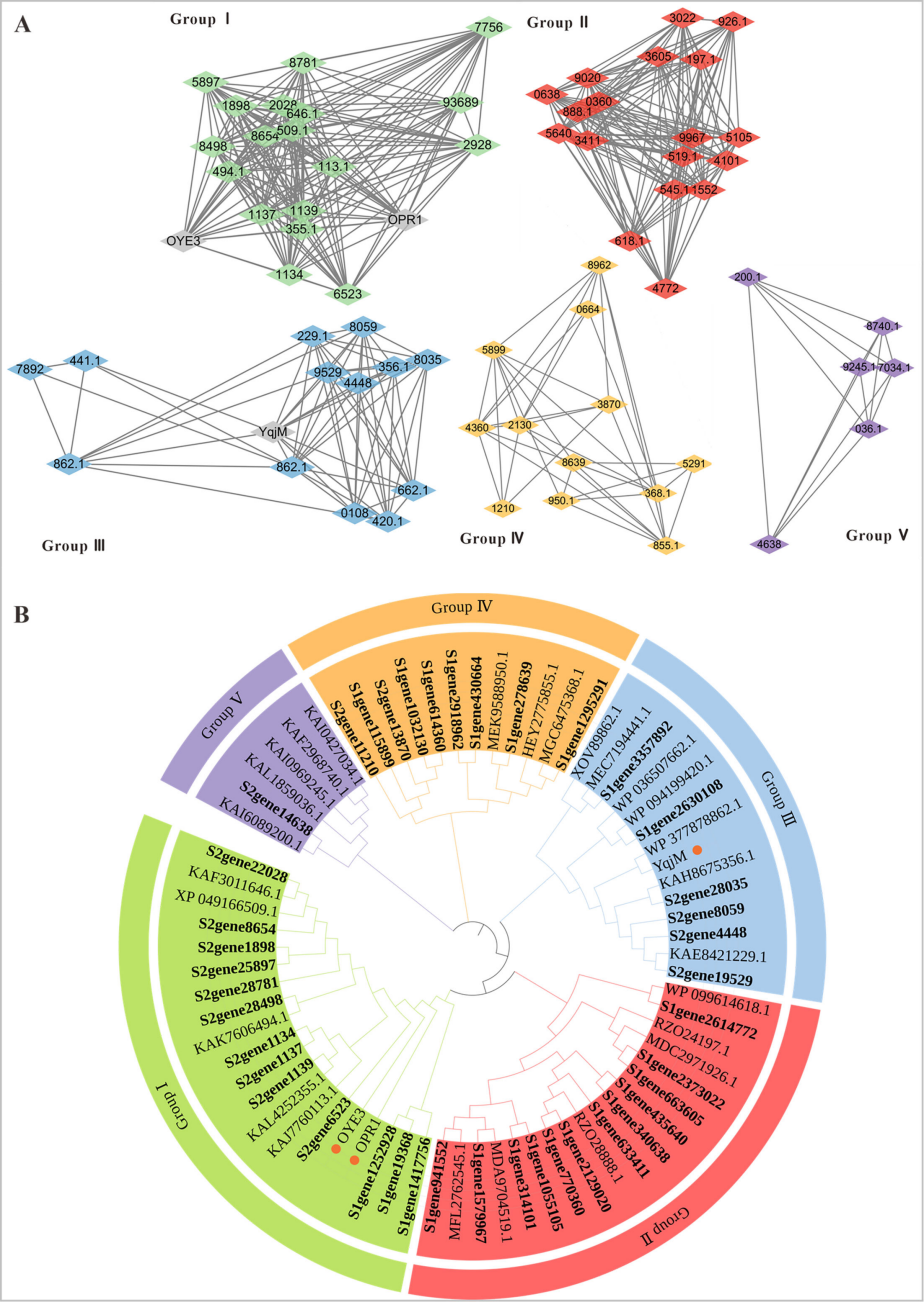
Mining of ERs. (**A**) ERs in sequence similarity networks (SSN); the probes (OPR1 [NCBI ID: NP_001234781.1], OYE3 [NCBI ID: NP_015154.1], and YjqM [NCBI ID: WP_255003398.1]) are marked with yellow. (**B**) Phylogenetic analysis of ERs, the probes (OPR1, OYE3, and YjqM) are marked with yellow dots. The bolded ERs correspond to those we mined from the South China Sea sediment metagenome, included in the supplemental material (Section 4).

By constructing sequence similarity networks (SSN) and phylogenetic trees (42, 43) (Fig. 2), we further assessed the diversity and evolutionary relationships among the 41 putative ERs. While most proteins exhibited relatively high sequence similarity and clustered closely, several did not co-aggregate with any reference clades, suggesting substantial divergence in sequence, structure, and potentially function. Notably, some enzymes displayed particularly low homology to the three probe sequences, implying their affiliation with previously unexplored branches of the ER family.

All 41 putative ene-reductase genes originating from the South China Sea deep-sea sediment metagenome were cloned into pET28a(+) and expressed in *E. coli* BL21(DE3) with an *N*-terminal His₆-tag (Fig. S2). Heterologous expression of extreme enzymes derived from marine metagenomes remains challenging due to their unique physicochemical adaptations (44, 45). To enhance soluble production, we implemented a series of optimization strategies. As summarized in Table S2, these efforts included modulation of induction conditions (e.g., temperature and IPTG concentration), fusion with various solubility-enhancing tags, and testing in alternative expression hosts. Ultimately, we succeeded in achieving soluble expression for 22 of the recombinant enzymes (Fig. S3), while the remaining 19 predominantly accumulated in inclusion bodies (SDS-PAGE analysis is shown in the supplemental material). Nevertheless, given that insolubility does not necessarily abolish catalytic function in crude environments, we evaluated the activity of all 41 enzymes directly from cell lysates without purification.

**Fig 3:**
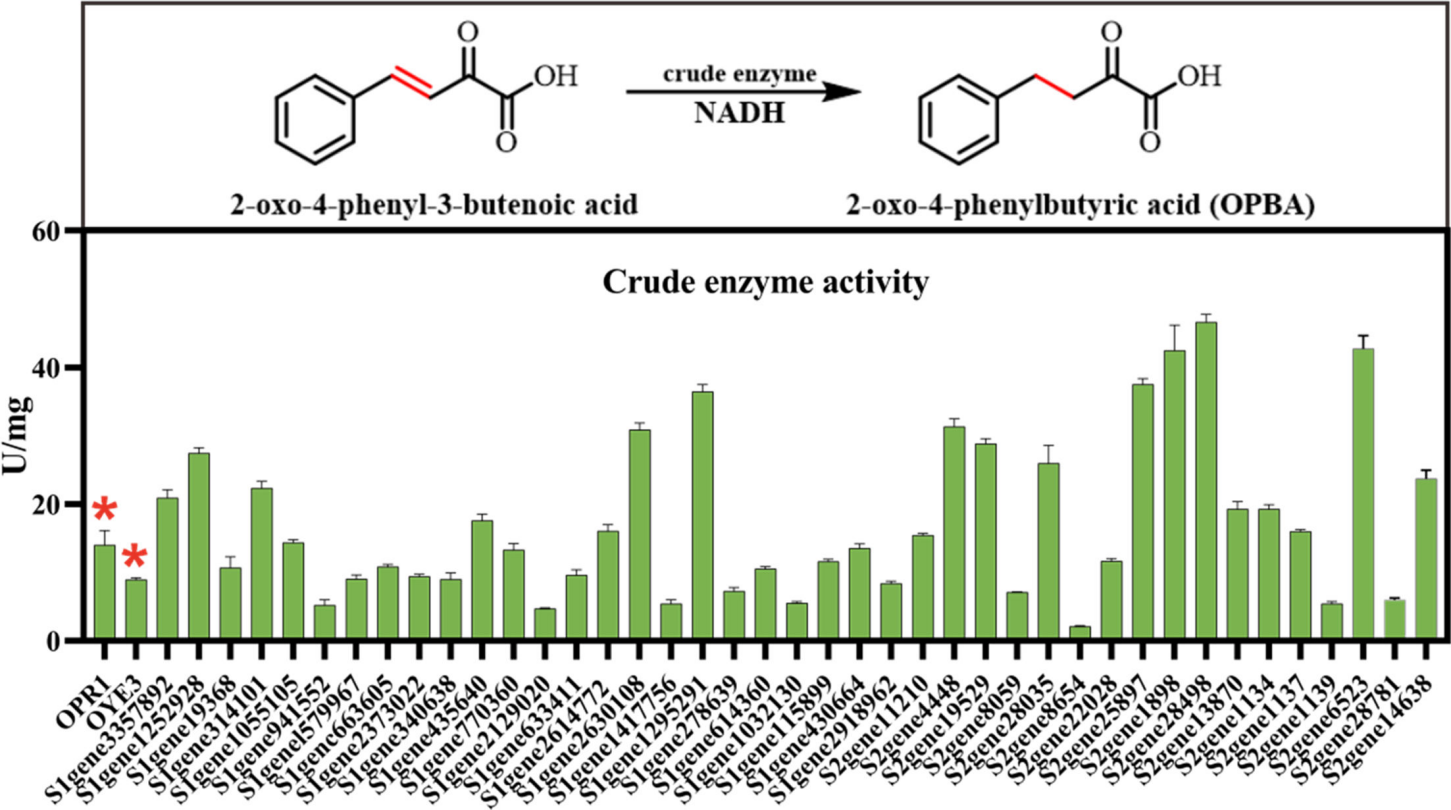
Catalytic activity screening of the recombinant ERs. Specific activities of crude lysates from the 41 recombinant ERs toward the model substrate 2-oxo-4-phenyl-3-butenoic acid. Activities are expressed as units per milligram of total protein (U/mg). One unit (U) of enzyme activity was defined as the amount of enzyme that consumes 1 μmol of NADH per minute. Reaction conditions: 30°C, 2 mM substrate, 0.2 mM NADH in 1 mL of PBS buffer (50 mM, pH 7.5). *Indicates the specific activities of the two reference enzymes, OPR1 and OYE3.

As shown in Fig. 3, crude lysates from all recombinant strains exhibited detectable activity toward the model substrate 2-oxo-4-phenyl-3-butenoic acid. Notably, approximately half of the 41 metagenomic-derived ene-reductases showed significantly higher activity than the two reference enzymes, OPR1 and OYE3, while the remaining half exhibited comparable levels. The activities of these enzymes ranged from 3 to 50 U/mg, with only one variant exhibiting activity lower than 5 U/mg. The majority demonstrated robust activity, with values concentrated around 20 U/mg, as measured using a standardized crude lysate with a total protein concentration of 1 mg/mL. These values meet common industrial thresholds for ene-reductase activity in shake-flask fermentations. For instance, Hunan Flag (HNFLAG) Biotechnology Co., Ltd. considers activities above 5 U/mg suitable for production-scale applications. This immediately highlights the potential of marine genetic resources for supplying readily applicable biocatalysts, reducing reliance on synthetic or mined catalysts.

**Fig 4:**
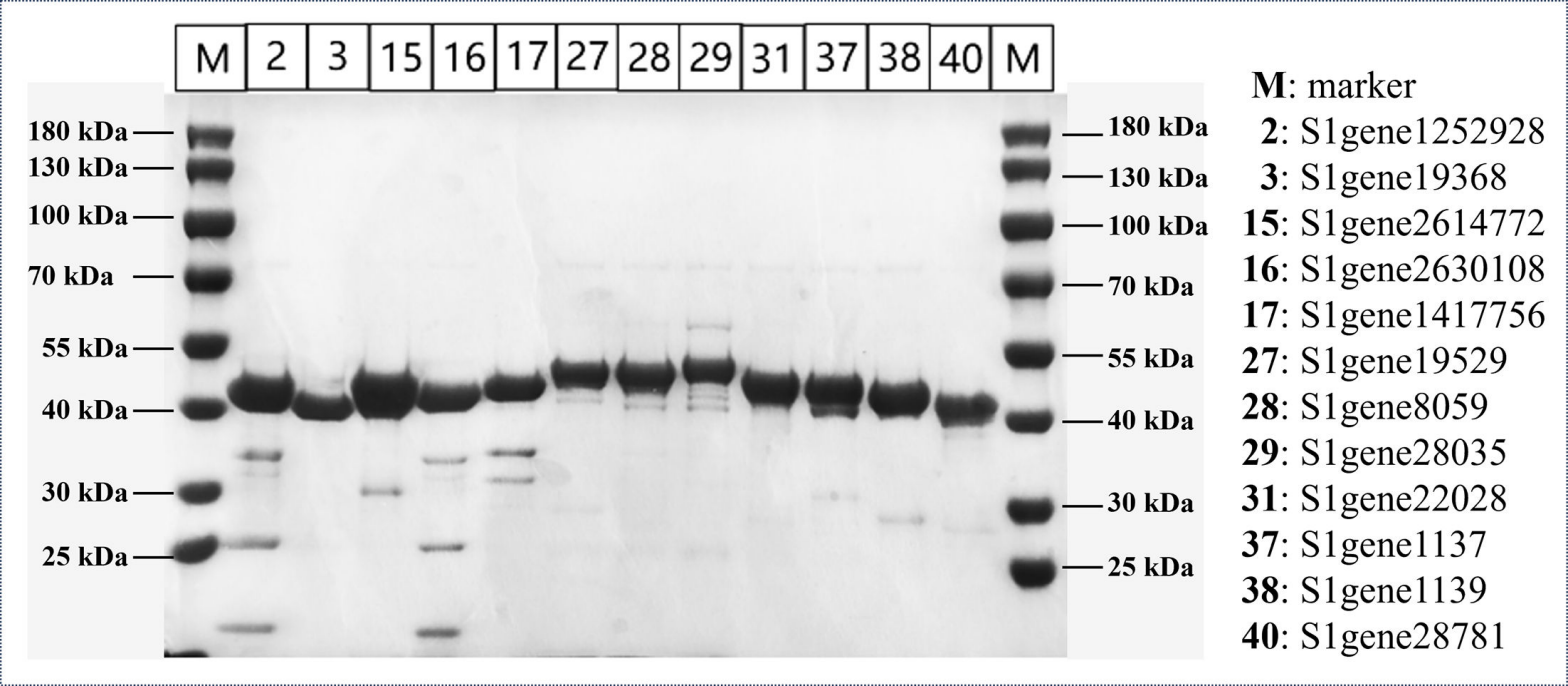
SDS-PAGE analysis of the representative 12 soluble ene-reductases in *E. coli* BL21(DE3).

To accurately determine the specific activities of the recombinant ene-reductases and minimize interference from native *E. coli* background activities (46), we selected 12 representative enzymes for purification. These candidates were chosen from the 22 solubly expressed enzymes based on high crude lysate activity and strong expression levels. As depicted in Fig. 4, all 12 purified enzymes showed clear, discrete bands at their anticipated molecular weights, indicating successful purification and minimal degradation. These results confirm not only the successful heterologous expression of these deep-sea sediment-derived enzymes in *E. coli*, but also attest to their structural integrity and significant biocatalytic potential, as further demonstrated in the subsequent catalytic studies.

### Biochemical characterization of the recombinant ERs: pH, temperature, substrate concentration, and cofactor dependence

To maximize the catalytic efficiency of the recombinant ERs obtained from the South China Sea deep-sea sediment metagenome, we systematically investigated the effects of key reaction parameters, including pH, temperature, cofactor specificity, and substrate concentration, on enzymatic activity. Characterization of these biochemical properties is particularly important for marine-derived enzymes, which often exhibit unique adaptations to extreme environments, such as enhanced stability under atypical pH and temperature conditions. Although our target substrate was 2-oxo-4-phenyl-3-butenoic acid, we used 4-phenyl-3-buten-2-one as a model substrate for initial characterization due to the generally low activity of these novel enzymes toward the target compound (which are discussed in detail in “Substrate scope of the purified ene-reductases,” below). Reductive activity was evaluated across a range of conditions: pH from 5.5 to 8.5, temperature from 15 to 40°C, and either NADH or NADPH as cofactor. Reactions were conducted with 10 mM substrate, and product formation was quantified by HPLC (see the supplemental material, part 1, for detailed data).

**Fig 5:**
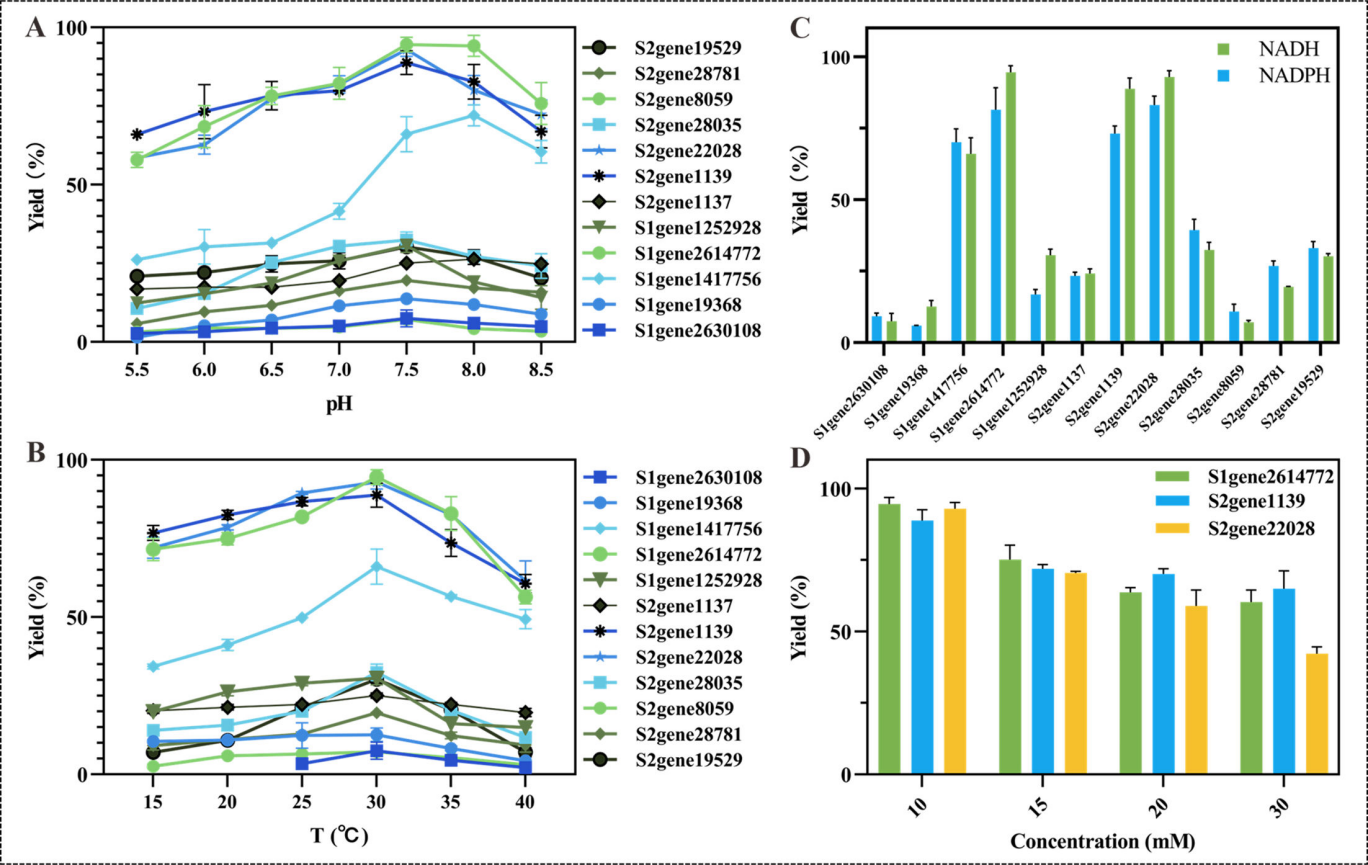
Effect of pH (**A**), temperature (**B**), cofactor (**C**), and substrate concentration (**D**) on enzyme activity of ERs. All reactions were performed with purified ERs at 1 mg/mL in 50 mM PBS buffer (pH 8.0) at 30°C for 16 h. The concentration of NAD(P)H was maintained at 1.2-fold molar excess relative to the substrate concentration in all assays. (**A**) Effect of pH on activity assayed with 10 mM substrate and 12 mM NADH. (**B**) Effect of temperature assayed with 10 mM substrate and 12 mM NADH. (**C**) Effect of cofactor assayed with 10 mM substrate and 12 mM NADH or NADPH. (**D**) Effect of substrate concentration (10, 20, and 30 mM) with 12, 24, and 36 mM NADH, respectively.

As shown in Fig. 5, the characterization of the 12 purified enzymes revealed properties highly desirable for a clean manufacturing process. Notably, most enzymes displayed highest activity at pH 7.5 in PBS buffer, although two variants exhibited optimal activity at pH 8.0. Notably, three enzymes (S2gene2614772, S2gene1139, and S2gene22028) showed exceptional performance, achieving nearly complete conversion of 10 mM substrate (Fig. 5A). Moreover, they retained significant activity under acidic and alkaline conditions (pH 5.5 and 8.5), converting ≥60% of the substrate, highlighting their tolerance to pH variation—a characteristic likely linked to their marine origin. The optimal temperature for the entire enzyme set was consistently 30°C (Fig. 5B). Again, S2gene2614772, S2gene1139, and S2gene22028 demonstrated remarkable cold adaptability, maintaining over 65% relative activity, even at 15°C, underscoring the potential cold-adapted nature of these deep-sea enzymes.

Cofactor preference assays revealed that all 12 purified enzymes could utilize both NADH and NADPH as electron donors (Fig. 5C). Although minor activity variations were observed between cofactors for certain enzymes, including S2gene2614772, S2gene1139, and S2gene22028, the differences were not substantial enough to indicate a strict cofactor preference.

To further examine substrate tolerance, we selected the three most active enzymes (S2gene2614772, S2gene1139, and S2gene22028), which achieved full conversion at 10 mM, and increased the substrate concentration to 30 mM (Fig. 5D). Unfortunately, complete conversion was not attained under these conditions, with yields plateauing between 50 and 70%. Consequently, we did not pursue higher substrate concentrations with the remaining nine enzymes, which already showed incomplete conversion at 10 mM.

Given the NAD(P)H-dependent nature of ERs, an efficient cofactor regeneration system is essential to sustain catalytic turnover and reduce operational costs. In this study, we employed a glucose dehydrogenase (bmGDH)-glucose system, one of the most widely adopted methods for *in situ* NADH regeneration, to support ER-catalyzed reductions (see Fig. S4 for details). The implementation of a glucose dehydrogenase (GDH)-glucose system for NADH regeneration ensured catalytic efficiency while minimizing cofactor waste, a key consideration for process economy and sustainability.

### Substrate scope of the purified ene-reductases

**Fig 6:**
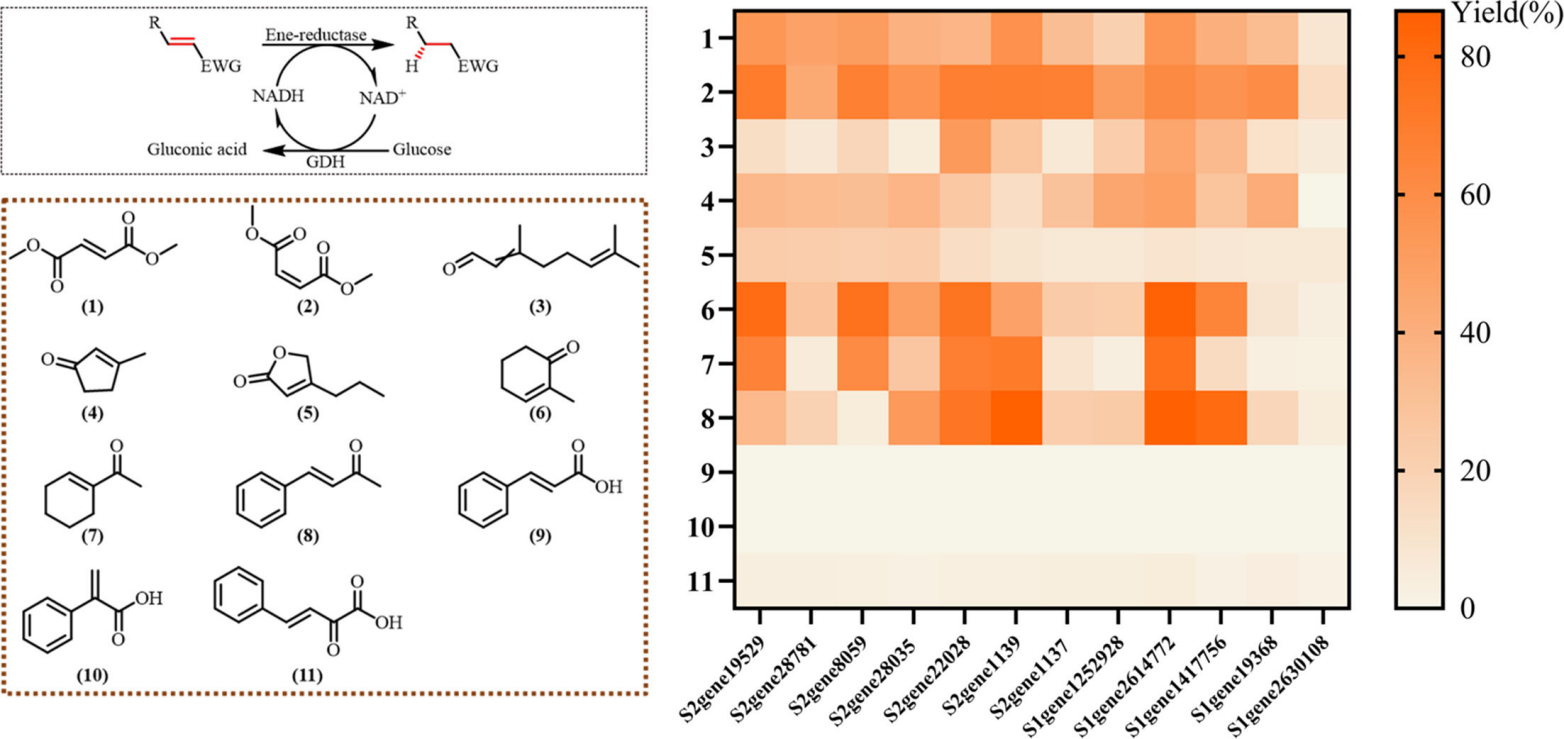
Substrate scope of the purified ene-reductases. Reaction conditions: substrate 10 mM, ERs 1 mg/mL, NADH regeneration system (0.25 mM NAD^+^, 50 mM glucose, and 0.25 mg/mL bmGDH) in PBS buffer (50 mM, pH of 7.5) for 16 h at 30°C.

With the purified enzymes and optimized reaction conditions established, we next evaluated the biocatalytic potential of the 12 ene-reductases for the asymmetric reduction of C=C bonds across a diverse substrate spectrum. Reactions were conducted in 1 mL PBS buffer (50 mM, pH 7.5) at 30°C using 10 mM substrate, an NADH regeneration system (0.25 mM NAD^+^, 50 mM glucose, and 0.25 mg/mL bmGDH), and 1 mg/mL purified enzyme for 16 h. A panel of 11 α,β-unsaturated compounds bearing electron-withdrawing groups was selected, encompassing esters (**1, 2, 5**), an aldehyde (**3**), ketones (**4, 6, 7, 8, 11**), and carboxylic acids (**9, 10, 11**) (Fig. 6).

As shown in Fig. 6 (and Fig. S5 through S15), the 12 ERs exhibited high activity toward substrates containing two electron-withdrawing groups. Excellent conversions were achieved for ester substrates, such as dimethyl fumarate (**1**) and dimethyl maleate (**2**), as well as ketone substrates, including 2-methyl-2-cyclohexen-1-one (**6**), 1-acetyl-1-cyclohexene (**7**), and benzalacetone (**8**). Among these, seven enzymes, including S1gene2614772, S2gene1139, and S2gene22028, demonstrated particularly high catalytic performance across multiple substrates.

Substrate activity was notably influenced by structural features. For ketone substrates (**4, 6, 7, 8, 11**), the substitution pattern markedly affected activity: several enzymes showed significantly higher conversion toward α-methyl-substituted cyclohexenone (**6**, up to 90% conversion) than toward β-methyl-substituted cyclopentenone (**4**), suggesting a possible preference for β-unsubstituted cyclic ketones. Acyclic enones, such as compound **7**, were also efficiently reduced.

In contrast, carboxylic acid substrates, including cinnamic acid (**9**), atropic acid (**10**), and especially the target substrate **11** (2-oxo-4-phenyl-3-butenoic acid), showed negligible conversion (5–10% yield) under all conditions. This result aligns with known variations in substrate specificity among ene-reductases from different sources and implies that α,β-unsaturated carboxylic acids may not be natural substrates for this enzyme family. It also offers a plausible explanation for the absence of prior reports on the biocatalytic reduction of compound **11** using ene-reductases despite the high value of its reduction product, 2-oxo-4-phenylbutyric acid (OPBA), as a pivotal prochiral intermediate in the synthesis of angiotensin-converting enzyme inhibitors.

While the wild-type ERs showed excellent activity toward various activated alkenes, they displayed negligible activity for the target substrate, 2-oxo-4-phenyl-3-butenoic acid (**11**), the direct precursor to OPBA (Fig. 6). This limitation is a common barrier in biocatalysis when moving from model compounds to structurally complex industrial substrates. Motivated by this challenge and the industrial significance of OPBA, we selected two representative enzymes for further protein engineering: S1gene1252928, which showed the highest native activity toward substrate **11** (albeit only ~10% conversion), and S2gene22028, which exhibited outstanding biochemical properties, including over 60% substrate conversion across a broad pH range (5.5–8.5) and retention of >65% relative activity at a low temperature of 15°C, along with near-quantitative conversion for most other substrates. Through directed evolution, we aimed to enable efficient biocatalytic reduction of **11** to OPBA, thereby expanding the synthetic utility of marine-derived ene-reductases.

### Protein engineering of ERs for enhanced synthesis of OPBA

Building on our previous success in expanding the substrate scope of valine dehydrogenases from a hot spring sediment metagenome through site-saturation mutagenesis, leading to engineered amino acid dehydrogenases (AADHs, EC 1.4.1.X) for efficient synthesis of non-natural *L*-amino acids and (*R*)-β-amino alcohols (26, 28), we applied a similar semi-rational strategy to enhance the activity of marine-derived ene-reductases toward the challenging substrate **11**.

**Fig 7:**
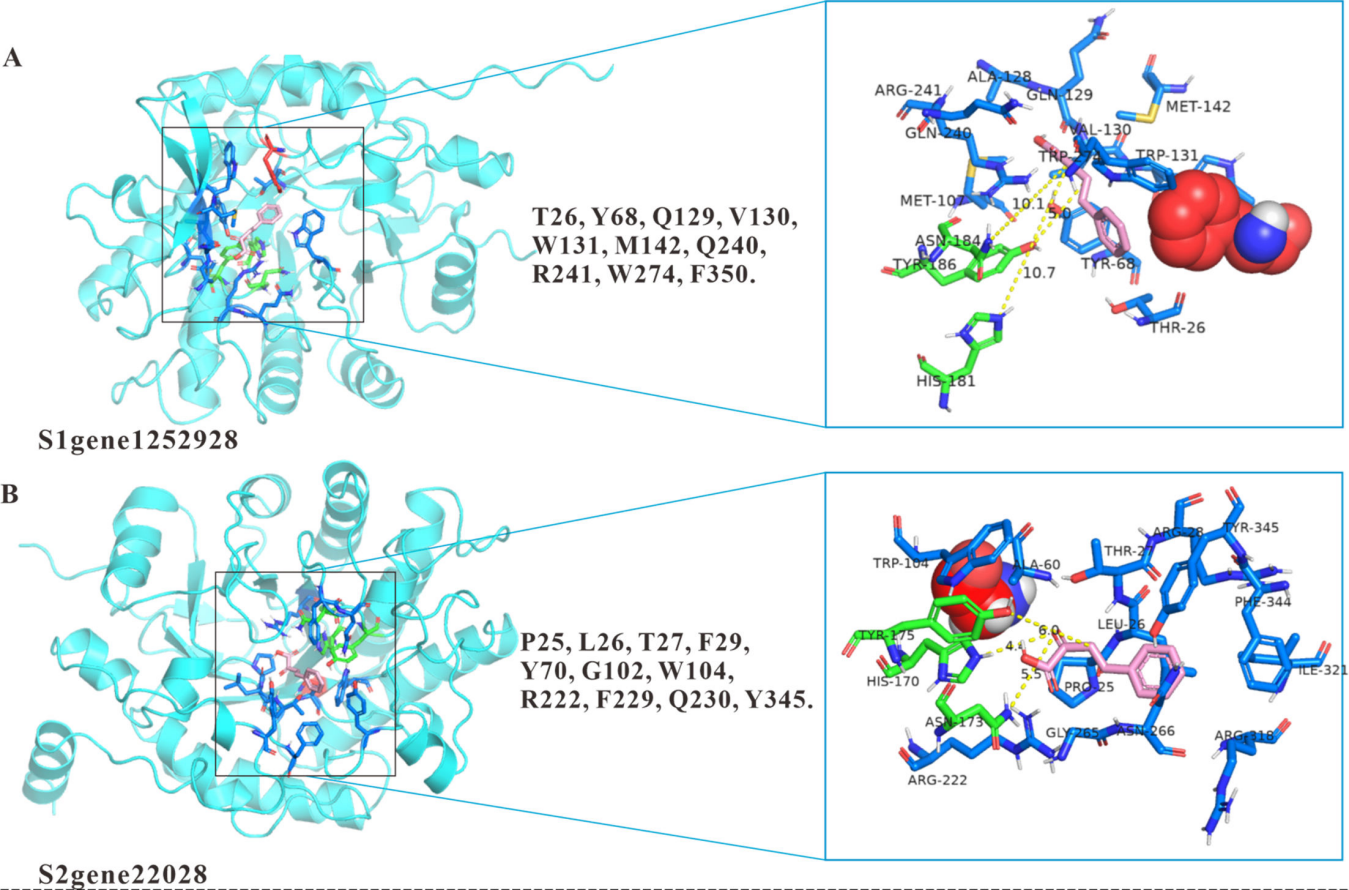
Molecular docking model diagram of the substrate 2-oxo-4-phenyl-3-butyric acid with ene reductases S1gene1252928 (**A**) and S2gene22028 (**B**) showing the interactions of residues in the enzyme active pocket within 5Å of the substrate. The F350 of S1gene1252928 and G102 of S2gene22028 are represented in red spheres. Pink indicates the substrate, and the substrate-binding sites and catalytic residues are marked in green.

The tertiary structures of S1gene1252928 and S2gene22028 were predicted using AlphaFold 2, and molecular docking with compound **11** was performed using AutoDock to identify residues potentially involved in substrate binding. Based on the docking results (Fig. 7), residues within 5 Å of the substrate were selected for alanine scanning mutagenesis to systematically evaluate their functional roles. A total of 10 residues (L26, Y68, Q129, V130, W131, M142, Q240, R241, W274, F350) in S1gene1252928 and 11 residues (P25, L26, T27, F29, Y70, G102, W104, R222, F229, G230, Y345) in S2gene22028 were mutated to alanine. Moreover, we have performed a structural superposition of S1gene1252928 and S2gene22028 and included it as Fig. S16 in the supplementary information. This figure illustrates the spatial alignment of their substrate-binding pockets and catalytic residues, providing insightful structural context for their functional differences.

**Fig 8:**
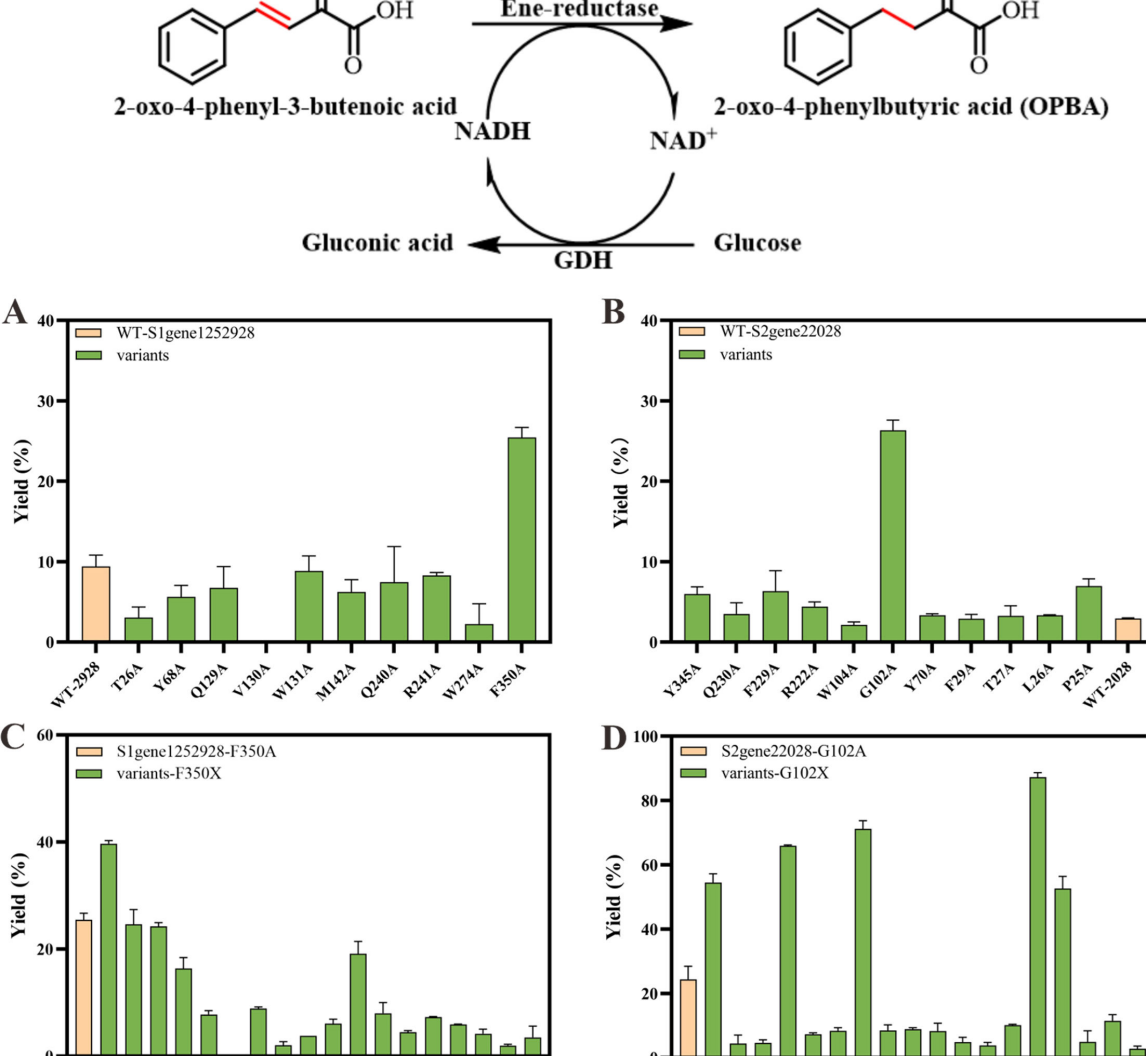
Directed evolution of S1gene1252928 and S2gene22028 and ER-mediated reduction reaction for the preparation of OPBA. (**A**) Results of ala-scanning mutagenesis of S1gene1252928. (**B**) Results of ala-scanning mutagenesis of S2gene22028. (**C**) Results of saturation mutagenesis screening of F350 in S1gene1252928. (**D**) Results of saturation mutagenesis screening of G102 in S2gene22028. Reaction conditions: 2-oxo-4-phenyl-3-butenoic acid 10 mM, ERs 1 mg/mL, NADH regeneration system (0.25 mM NAD^+^, 50 mM glucose, and 0.25 mg/mL bmGDH) in PBS buffer (50 mM, pH of 7.5) for 24 h at 30°C.

We first performed ala-screening to construct several mutants, including T26A, Y68A, Q129A, V130A, W131A, M142A, Q240A, R241A, W274A, and F350A, for S1 gene 1252928 (Fig. 8A) and P25A, L26A, T27A, F29A, Y70A, G102A, W104A, R222A, F229A, Q230A, and Y345A for S2 gene 22028 (Fig. 8B). The catalytic activity regarding the production of OPBA clearly revealed that the F350A mutant of S1gene1252928 and the G102A mutant of S2gene22028 increased the catalytic yield by 2.7- and 9.0-fold compared to the wild-type enzymes, respectively (Fig. 8A and B), indicating their critical role in substrate recognition or catalysis. This result is consistent with recent studies on ene-reductase engineering. For instance, Wang and co-workers (6) demonstrated that mutating W104 to alanine within 5 Å of the substrate binding site in SeER from *Saccharomyces eubayanus* significantly enhanced stereochemical selectivity toward β-cyano cinnamic esters. Similarly, mutation of W116 to isoleucine in the substrate-binding pocket of OYE3 led to improved catalytic activity (47). These findings collectively support the strategy of reshaping the substrate-binding pocket to enhance the catalytic performance of ene-reductases.

To further optimize catalytic efficiency, saturation mutagenesis was conducted at positions F350 (S1gene1252928) and G102 (S2gene22028). Among all variants, the G102S mutant of S2gene22028 delivered the most substantial improvement, achieving 90% conversion at 10 mM substrate concentration, a 30-fold increase over the wild-type enzyme (Fig. 8C and D). This successful engineering demonstrates that the catalytic repertoire of natural enzymes can be rationally expanded to address specific synthetic challenges, thereby enabling green alternatives to entrenched chemical methods.

### Scale-up preparation of OPBA and evaluation of industrial applicability

The ultimate test of a cleaner production strategy is its scalability and practical performance. Hence, the S2gene22028-G102S variant was employed for the scale-up biocatalytic synthesis of OPBA. In this system, 2-oxo-4-phenyl-3-butenoic acid served as the substrate at a final concentration of 100 mM in a 50 mL reaction volume. To sustain coenzyme cycling, the reaction was coupled to a glucose dehydrogenase (GDH)-glucose coenzyme regeneration module, which enabled efficient *in situ* regeneration of NADH. Under these optimized conditions, transformation of 5 mmol 2-oxo-4-phenyl-3-butenoic acid (**11**) could be achieved, with no inhibition observed, giving the target product OPBA with a catalytic yield of 62%, corresponding to a final product concentration of approximately 11 mg/mL and a space-time yield of 0.55 g·L⁻¹·h⁻¹. The yield reported is based on analytical conversion as determined by HPLC using a calibration curve derived from an authentic OPBA standard.

Notably, the product concentration attained in this scale-up study meets established industrial standards for biocatalytic processes; for instance, Hunan Flag (HNFLAG) Biotechnology Co., Ltd (China) considers product tiers above 10–20 mg/mL economically viable for production-scale applications.

### Conclusion

In this study, we successfully mined the deep-sea sediment metagenome of the South China Sea and identified 41 putative ene-reductase (ER) genes, among which 22 were solubly expressed in *E. coli*. Biochemical characterization revealed that these marine-derived ERs possess broad substrate promiscuity, efficiently reducing eight conventional α,β-unsaturated compounds with conversions up to 90%. Notably, three enzymes (S2gene2614772, S2gene1139, and S2gene22028) exhibited exceptional environmental adaptability, maintaining high catalytic activity across a broad pH range (5.5–8.5) and at low temperatures (as low as 15°C), underscoring their robustness and potential for industrial applications under non-conventional conditions.

Notably, we addressed a significant biocatalytic gap by targeting the reduction of 2-oxo-4-phenyl-3-butenoic acid, a prochiral precursor to angiotensin-converting enzyme inhibitors (ACEIs) that had not been previously reduced by any known ene-reductase. While the wild-type enzymes showed minimal activity toward this sterically hindered substrate, structure-guided engineering of two promising candidates (S1gene1252928 and S2gene22028) led to the identification of key residues influencing substrate binding and catalysis. Through alanine scanning and saturation mutagenesis, we obtained the mutant S2gene22028-G102S, which exhibited a 30-fold increase in activity and achieved 90% conversion at 10 mM substrate concentration.

Scale-up biocatalytic synthesis using the G102S mutant was conducted with 5 mmol of the substrate 2-oxo-4-phenyl-3-butenoic acid (100 mM in 50 mL reaction system), demonstrating industrial relevance. The process yielded OPBA at a concentration of 11 mg/mL with 62% conversion, meeting commercial viability thresholds. These results not only underscore the South China Sea as an underexplored reservoir of novel and robust biocatalysts but also validate the integration of metagenomic mining and protein engineering as a powerful strategy for developing enzymatic routes to high-value pharmaceutical intermediates.

## Data Availability

The amino acid sequences of the 41 putative ene-reductases identified in this study have been deposited in the NCBI database with accession numbers PX562690 to PX562730.

## SUPPLEMENTAL MATERIAL

Supplemental material aem.02333-25-s0001.pdf Supplemental methods, Tables S1 and S2, Fig. S1 to S16, and amino acid sequences of the mined ERs.

ASM does not own the copyrights to Supplemental Material that may be linked to, or accessed through, an article. The authors have granted ASM a non-exclusive, world-wide license to publish the Supplemental Material files. Please contact the corresponding author directly for reuse.
