# Supplementary material for: Mining and engineering of ene-reductases from marine sediment metagenome for prochiral ACE inhibitor synthesis
Source: Appl Environ Microbiol. 2026 Jan 15;92(2):e02333-25. doi: 10.1128/aem.02333-25 (PMC12915312; doi:10.1128/aem.02333-25)
Supplement: Supplemental material — Supplemental methods, Tables S1 and S2, Fig. S1 to S16, and amino acid sequences of the mined ERs. [file aem.02333-25-s0001.pdf]

Supporting Information

Mining and engineering of ene-reductases from marine sediment metagenome for prochiral ACE inhibitor synthesis

Yating Zou,[a] Jinghui Zhou,[b] Yongyi Zeng,[a] Bishuang Chen,[a,c]* Lan Liu,[a,c] Gang Xu[b]

[a] School of Marine Sciences, Sun Yat-Sen University, Zhuhai 519080, China.

[b] Hunan Flag Biotechnology Co., Ltd., Changsha 410000, China.

[c] Southern Marine Science and Engineering Guangdong Laboratory (Zhuhai), Zhuhai 519080, China.

*Corresponding Authors

Bishuang Chen: chenbsh23@mail.sysu.edu.cn

Table of Contents

1. **Analytical Method (HPLC and GC)**
   1.1 Analytical data for characterizing the reduction of 1, 2, 4, 6, 7
   1.2 Analytical data for characterizing the reduction of 3
   1.3 Analytical data for characterizing the reduction of 5
   1.4 Analytical data for characterizing the reduction of 8
   1.5 Analytical data for characterizing the reduction of 9
   1.6 Analytical data for characterizing the reduction of 10
   1.7 Analytical data for characterizing the reduction of 11

2. **Supplementary tables**
   Table S1. Primers used for this study
   Table S2. Different strategies to improve soluble expression of putative ene-reductases

3. **Supplementary Figures**
   Figure S1. Sequence alignment of OPR1, OYE3, YjqM with the other 41 ERs.
   Figure S2. SDS-PAGE analysis of overexpression of 41 novel ERs in *E. coli* BL(21)DE3.
   Figure S3. SDS-PAGE analysis confirmed that 22 recombinant ERs were successfully expressed in a soluble form.
   Figure S4. Analysis of optimal reaction conditions for coenzyme cycling.
   Figure S5-S15. Representative GC chromatogram of all compounds used in this study.
   Figure S16. A structural superposition of S1gene1252928 and S2gene22028

4. **Amino acid sequences of the mined ERs**
   The original amino acid sequences of 41 ERs.

1 Analytical Method (HPLC and GC)

1.1 Analytical data for characterizing the reduction of 1, 2, 4, 6, 7:

GC (Restek10224 Rtx-5, 30 m × 0.32 mm × 0.25 µm, N_2): Initial temperature 90 °C; then ramped at 1 °C/min to 100 °C and held for 1 min; finally ramped at 5 °C/min to 130 °C.

Tr (**1**) = 3.55 min, tr (product) = 4.24 min; tr (**2**) = 4.07 min, tr (product) = 4.24 min; tr (**4**) = 3.34 min, tr (product) = 2.3 min; tr (**6**) = 3.77 min, tr (product) = 3.14 min; tr (**7**) = 6.14 min, tr (product) = 4.38 min.

1.2 Analytical data for characterizing the reduction of 3:

GC (Restek10224 Rtx-5, 30 m × 0.32 mm × 0.25 µm, N_2): Initial temperature 80 °C; then ramped at 1 °C/min to 95 °C and held for 1 min; followed by ramping at 0.5 °C/min to 98 °C and held for 1 min; finally ramped at 20 °C/min to 150 °C.

Tr (**3**) = 16.18 min and 18.59 min, tr (product) = 10.56 min.

1.3 Analytical data for characterizing the reduction of 5:

GC (Restek10224 Rtx-5, 30 m × 0.32 mm × 0.25 µm, N_2): Initial temperature 80 °C; then ramped at 20 °C/min to 115 °C and held for 1 min; followed by ramping at 6 °C/min to 145 °C and held for 2 min; finally ramped at 30 °C/min to 200 °C.

Tr (**5**) = 6.92 min, tr (product) = 5.65 min.

1.4 Analytical data for characterizing the reduction of 8:

GC (Restek10224 Rtx-5, 30 m × 0.32 mm × 0.25 µm, N_2): Initial temperature 40 °C; then ramped at 7 °C/min to 180 °C and held for 1 min; finally ramped at 20 °C/min to 220 °C.

Tr (**8**) = 15.97 min, tr (product) = 13.53 min.

1.5 Analytical data for characterizing the reduction of 9:

HPLC (Agilent 2.7 µm InfinityLab Poroshell 120 Phenyl-Hexyl, 4.6 x 250 mm): 210 nm and 254 nm, 0.6 mL/min, 35°C, A: H_2O+0.1% HAc, B: ACN; 78%A, 22%B, 40 min.

Tr (**9**) = 34.28 min, tr (product) = 30.66 min.

1.6 Analytical data for characterizing the reduction of 10:

HPLC (Agilent 2.7 µm InfinityLab Poroshell 120 Phenyl-Hexyl, 4.6 x 250 mm): 210 nm, 0.6 mL/min, 35°C, A: H_2O+0.1% HAc, B: ACN; gradient: 92%A, 8%B -> 70%A, 30%B, 60 min.

Tr (**10**) = 47.30 min, tr (product) = 48.52 min.

1.7 Analytical data for characterizing the reduction of 11:

HPLC (Agilent 2.7 µm InfinityLab Poroshell 120 Phenyl-Hexyl, 4.6 x 250 mm): 210 nm and 280 nm, 0.6 mL/min, 35°C, A: H_2O+0.1% HAc, B: ACN; 79%A, 21%B, 30 min.

Tr (**11**) = 14.86 min, tr (product) = 18.14 min.

2 Supplementary tables

Table S1. Primers used for this study

**Primers used for ala-scanning mutagenesis of S1gene1252928**	
Primers	Ssequences (5'to 3')[a]
T26A-F	ACCGATGGCGCGTGCACGTACCAC
T26A-R	GTGCACGCGCCATCGGTGCCAGTG
Y68A-F	AAAGGTGCGTCTTTCACTCCGGGTATC
Y68A-R	GAAAGACGCACCTTTACCCTGAGAGCTG
M107A-F	GGTCGTGCGAGCCACGAAAGCCTTCATG
M107A-R	CTTTCGTGGCTCGCACGACCAACGTGC
Q129A-F	GATGCTGCGGTTTGGATCGTTGATCCG
Q129A-R	CCAAACCGCAGCATCTGGAGACAGAG
V130A-F	ATGCTCAGGCGTGGATCGTTGATCC
V130A-R	CGATCCACGCCTGAGCATCTGGAGACAG
W131A-F	CAGGTTGCGATCGTTGATCCGGAAACTG
W131A-R	AACGATCGCAACCTGAGCATCTGGAGAC
M142A-F	GGTGGTGCGGTGGATTGCCCAGTAC
M142A-R	ATCCACCGCACCACCTTCACCAGTTTC
Q240A-F	ATCACTGCGCGTAACATGGCAGACG
Q240A-R	GTTACGCGCAGTGATGTACGGAGACAG
R241A-F	ACTCAGGCGAACATGGCAGACGATG
R241A-R	CATGTTCGCCTGAGTGATGTACGGAGAC
W274A-F	GCTGACGCGGACGACGCACCGATC
W274A-R	GTCGTCCGCGTCAGCTTCAGACAGATGGATG
F350A-F	TACTCTGGCGGGCGGTACTGAAGCTGG
F350A-R	ACCGCCCGCCAGAGTACCCGGATC

**Primers used for ala-scanning mutagenesis of S2gene22028**	
Primers	Ssequences (5'to 3')[a]
P25A-F	GCTATGGCTGCGCTGACTCGTTTCCG
P25A-R	ACGAGTCAGCGCAGCCATAGCGATAC
L26A-F	ATGGCTCCGGCGACTCGTTTCCGTG
L26A-R	GAAACGAGTCGCCGGAGCCATAGCGATACG
T27A-F	CTCCGCTGGCGCGTTTCCGTGCAGAC
T27A-R	ACGGAAACGCGCCAGCGGAGCCATAG
F29A-F	CTGACTCGTGCGCGTGCAGACGAGAAC
F29A-R	TCTGCACGCGCACGAGTCAGCGGA
Y70A-F	GGGCGGTGCGGGTCTGGTTCCGGGTA
Y70A-R	ACCAGACCCGCACCGCCCGCTTTCTTAG
G102A-F	ATGTTTCTGGCGCTGTGGGCACTGGGTC

G102A-R	TGCCCACAGCGCCAGAAACATAGAGCTG
W104A-F	TGCAGCTGGCGGCACTGGGTCGTGTTG
W104A-R	ACCCAGTGCCGCCAGCTGCAGAAACATAG
R222A-F	GTGGGCATGGCGCTGTCTCCGTTCTC
R222A-R	CGGAGACAGCGCCATGCCCACTTTGC
F229A-F	TTCTCTGACGCGCAGGGTATGCACATG
F229A-R	CATACCCTGCGCGTCAGAGAACGGAGAC
G230A-F	TCTGACTTCGCGGGTATGCACATGGCAG
G230A-R	GTGCATACCCGCGAAGTCAGAGAACGG
Y345A-F	GATTTGTTCGCGAACGCTGGTGAGAAGAAAGG
Y345A-R	ACCAGCGTTCGCGAACAAATCACGGTC

Primers used for saturation mutagenesis of S1gene1252928

Primers	Ssequences (5'to 3')[a]
F350C-F	GTACTCTGTGCGGCGGTACTGAAGCTGGTTAC
F350C-R	GTACCGCCGCACAGAGTACCCGGATCAAATTC
F350D-F	GTACTCTGGATGGCGGTACTGAAGCTGGTTAC
F350D-R	GTACCGCCATCCAGAGTACCCGGATCAAATTC
F350E-F	GTACTCTGGAAGGCGGTACTGAAGCTGGTTAC
F350E-R	GTACCGCCTTCCAGAGTACCCGGATCAAATTC
F350G-F	GTACTCTGGGCGGCGGTACTGAAGCTGGTTAC
F350G-R	GTACCGCCGCCCAGAGTACCCGGATCAAATTC
F350H-F	GTACTCTGCATGGCGGTACTGAAGCTGGTTAC
F350H-R	GTACCGCCATGCAGAGTACCCGGATCAAATTC
F350I-F	GGTACTCTGATTGGCGGTACTGAAGCTGGTTAC
F350I-R	AGTACCGCCAATCAGAGTACCCGGATCAAATTC
F350K-F	GGTACTCTGAAAGGCGGTACTGAAGCTGGTTAC
F350K-R	CAGTACCGCCTTTCAGAGTACCCGGATCAAATTC
F350L-F	GTACTCTGCTGGGCGGTACTGAAGCTGGTTAC
F350L-R	GTACCGCCCAGCAGAGTACCCGGATCAAATTC
F350M-F	GTACTCTGATGGGCGGTACTGAAGCTGGTTAC
F350M-R	GTACCGCCCATCAGAGTACCCGGATCAAATTC
F350N-F	GTACTCTGAACGGCGGTACTGAAGCTGGTTAC
F350N-R	GTACCGCCGTTCAGAGTACCCGGATCAAATTC
F350P-F	GTACTCTGCCGGGCGGTACTGAAGCTGGTTAC
F350P-R	GTACCGCCCGGCAGAGTACCCGGATCAAATTC
F350Q-F	GTACTCTGCAGGGCGGTACTGAAGCTGGTTAC
F350Q-R	GTACCGCCCTGCAGAGTACCCGGATCAAATTC
F350R-F	GTACTCTGCGCGGCGGTACTGAAGCTG
F350R-R	GTACCGCCGCGCAGAGTACCCGGATCAAATTC
F350S-F	GTACTCTGAGCGGCGGTACTGAAGCTGGTTAC
F350S-R	GTACCGCCGCTCAGAGTACCCGGATCAAATTC
F350T-F	GTACTCTGACCGGCGGTACTGAAGCTGGTTAC

F350T-R	GTACCGCCGGTCAGAGTACCCGGATCAAATTC
F350V-F	GTACTCTGGTGGGCGGTACTGAAGCTGGTTAC
F350V-R	GTACCGCCCACCAGAGTACCCGGATCAAATTC
F350W-F	GTACTCTGTGGGGCGGTACTGAAGCTGGTTAC
F350W-R	GTACCGCCCCACAGAGTACCCGGATCAAATTC
F350Y-F	GGGTACTCTGTATGGCGGTACTGAAGCTGGTTAC
F350Y-R	CAGTACCGCCATACAGAGTACCCGGATCAAATTC

Primers used for saturation mutagenesis of S2gene22028

Primers	Ssequences (5'to 3')[a]
G102C-F	ATGTTTCTGTGTCTGTGGGCACTGGGTC
G102C-R	TGCCCACAGACACAGAAACATAGAGCTGC
G102D-F	ATGTTTCTGGATCTGTGGGCACTGGGTC
G102D-R	TGCCCACAGATCCAGAAACATAGAGCTGC
G102E-F	ATGTTTCTGGAACTGTGGGCACTGGGTC
G102E-R	TGCCCACAGTTCCAGAAACATAGAGCTGC
G102F-F	ATGTTTCTGTTTCTGTGGGCACTGGGTCG
G102F-R	TGCCCACAGAAACAGAAACATAGAGCTGCC
G102H-F	ATGTTTCTGCATCTGTGGGCACTGGGTC
G102H-R	TGCCCACAGATGCAGAAACATAGAGCTGC
G102I-F	ATGTTTCTGATTCTGTGGGCACTGGGTCG
G102I-R	TGCCCACAGAATCAGAAACATAGAGCTGCC
G102K-F	ATGTTTCTGAAGCTGTGGGCACTGGGTC
G102K-R	TGCCCACAGCTTCAGAAACATAGAGCTGC
G102L-F	ATGTTTCTGCTTCTGTGGGCACTGGGTC
G102L-R	TGCCCACAGAAGCAGAAACATAGAGCTGC
G102M-F	ATGTTTCTGATGCTGTGGGCACTGGGTC
G102M-R	TGCCCACAGCATCAGAAACATAGAGCTGC
G102N-F	ATGTTTCTGAATCTGTGGGCACTGGGTCG
G102N-R	TGCCCACAGATTCAGAAACATAGAGCTGCC
G102P-F	ATGTTTCTGCCTCTGTGGGCACTGGGTC
G102P-R	TGCCCACAGAGGCAGAAACATAGAGCTG
G102Q-F	ATGTTTCTGCAACTGTGGGCACTGGGTC
G102Q-R	TGCCCACAGTTGCAGAAACATAGAGCTG
G102R-F	ATGTTTCTGAGACTGTGGGCACTGGGTC
G102R-R	TGCCCACAGTCTCAGAAACATAGAGCTGC
G102S-F	ATGTTTCTGTCTCTGTGGGCACTGGGTC
G102S-R	TGCCCACAGAGACAGAAACATAGAGCTGC
G102T-F	ATGTTTCTGACTCTGTGGGCACTGGGTC
G102T-R	TGCCCACAGAGTCAGAAACATAGAGCTGC
G102V-F	ATGTTTCTGGTTCTGTGGGCACTGGGTC
G102V-R	TGCCCACAGAACCAGAAACATAGAGCTGC
G102W-F	ATGTTTCTGTGGCTGTGGGCACTGGGTC

G102W-R	TGCCCACAGCCACAGAAACATAGAGCTG
G102Y-F	ATGTTTCTGTATCTGTGGGCACTGGGTCG
G102Y-R	TGCCCACAGATACAGAAACATAGAGCTGCC

a. underlined codon encodes desired amino acid substitution.

Table S2. Different strategies to improve soluble expression of putative ene-reductases

Attempt	Modification	Reason
1	Combinations of different temperatures, IPTG concentrations and incubation times	Slower expression could lead to more soluble enzyme
2	Co-expression with all different chaperones (MBP, LSSA12, HfYFP and GST) set at different temperatures	Chaperones can help with protein folding
3	Expression in *E. coli* BL21 (DE3) Rosetta2 cells	Rosetta2 contains tRNAs for rare codons in *E. coli*
4	Expression in pBAD-His plasmid at different arabinose concentrations and temperatures	Better control of expression level using arabinose
5	Resolubilisation of inclusion bodies	Trying to resollubilise the large amount of protein present in insoluble fraction
6	Expression without His-tag	His-tag might impair proper folding
7	Expression in low copy plasmid	Less expression can improve folding

3. Supplementary figures

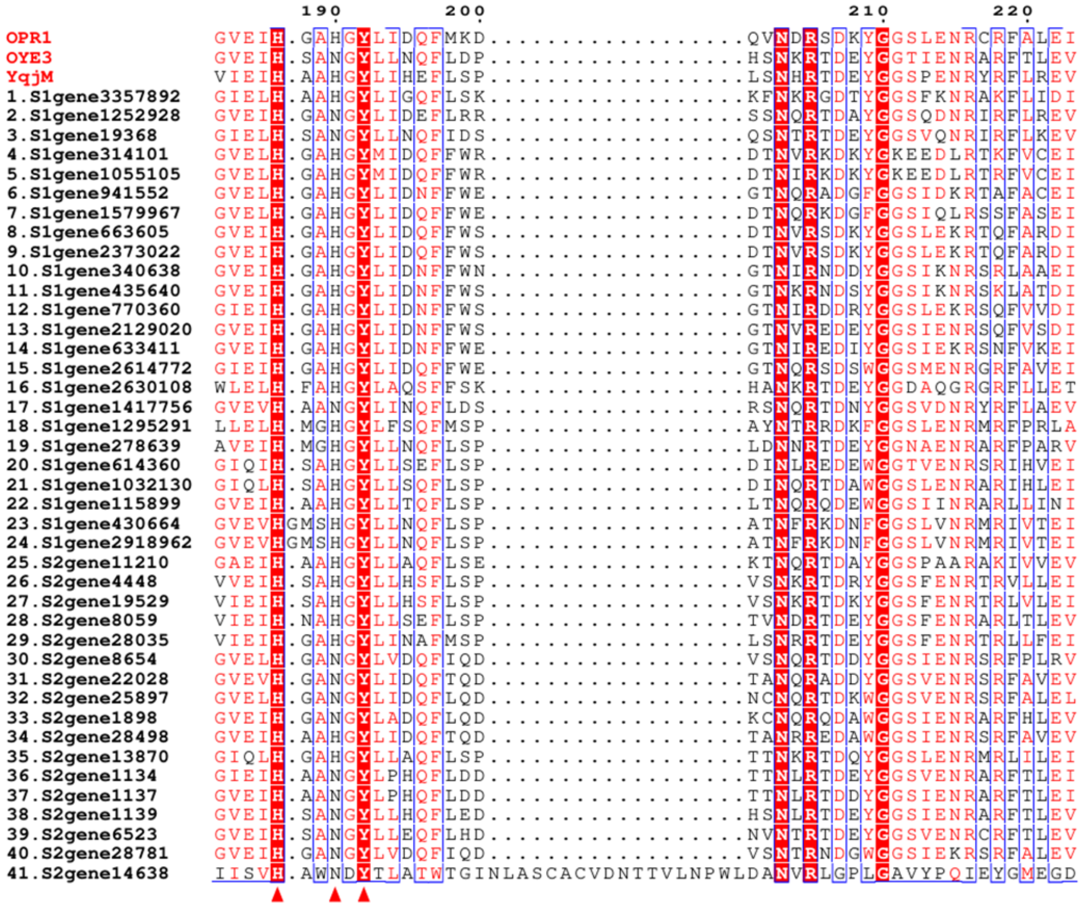

Figure S1. Sequence alignment of OPR1, OYE3, YjqM with the other 41 ERs. Substrate binding sites and catalytic residue are marked with red triangles.

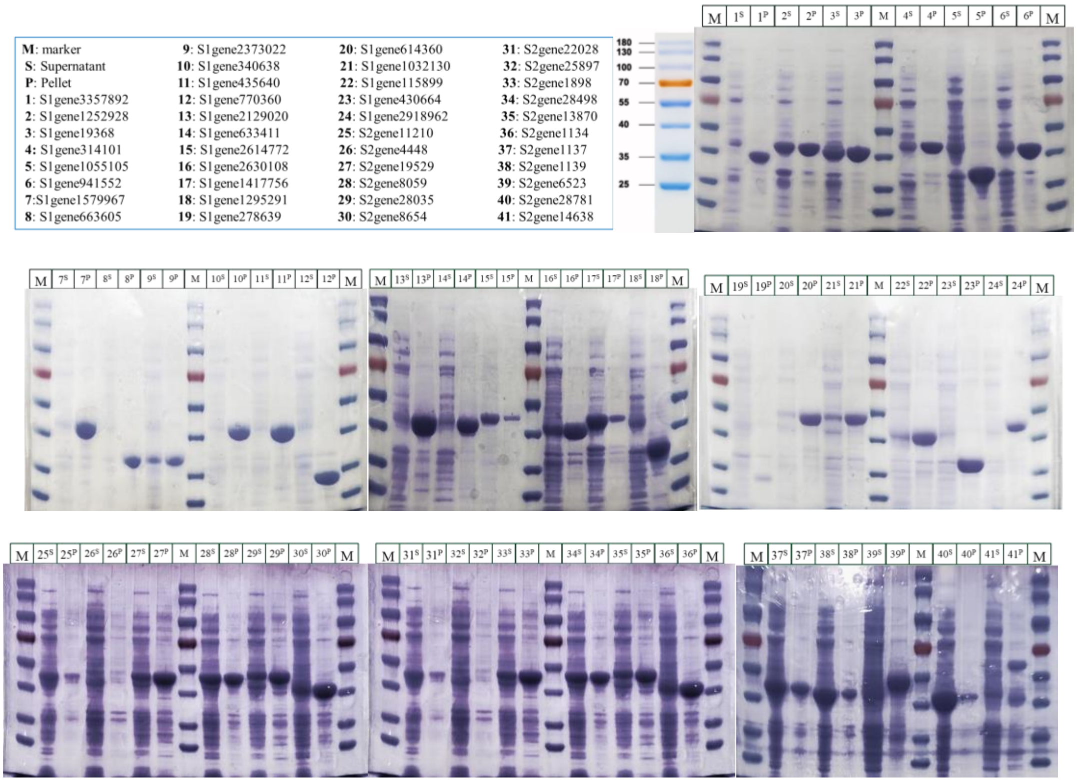

Figure S2. SDS-PAGE analysis of overexpression of 41 novel ERs in *E. coli* BL(21)DE3.

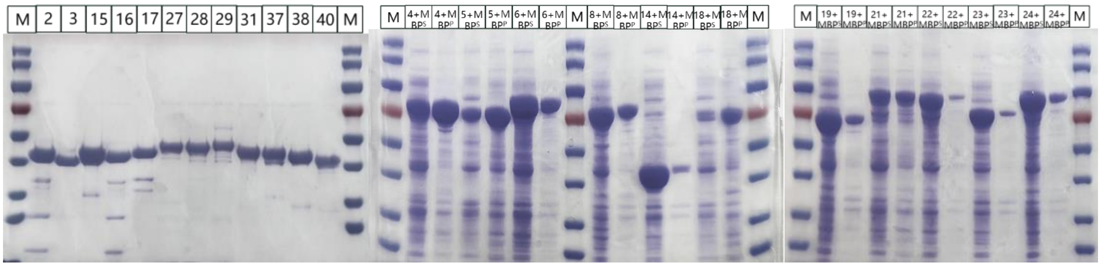

Figure S3. SDS-PAGE analysis confirmed that 22 recombinant ERs were successfully expressed in a soluble form.

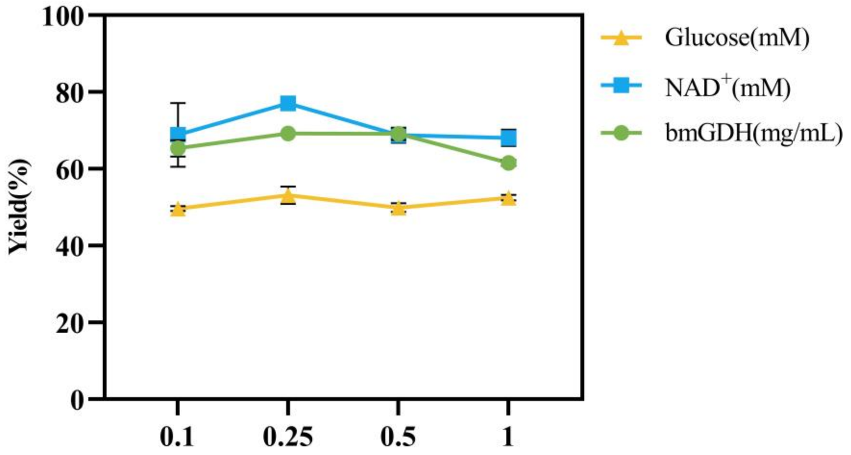

Figure S4. Analysis of optimal reaction conditions for coenzyme cycling. Condition: substrate (4-phenyl-3-buten-2-one) 10 mM, bmGDH 1 mg/mL, NAD^+ 0.5 mM, glucose 100 mM in PBS buffer (50 mM, pH of 8.0) for 16 h at 30 °C. Optimal reaction condition: bmGDH 0.25 mg/mL, NAD^+ 0.25 mM, glucose 50 mM.

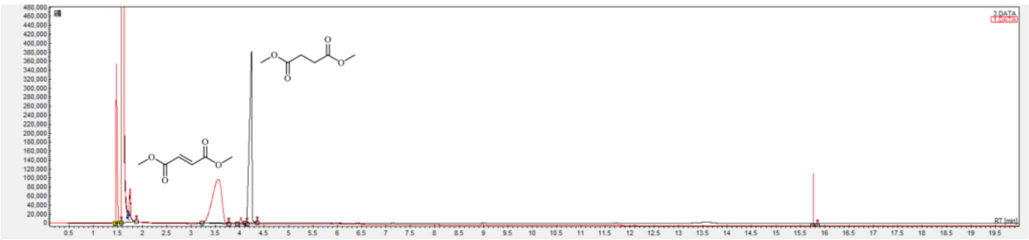

Figure S5. Representative GC chromatogram of **1**. Tr(**1**) = 3.55 min, tr(product) = 4.24 min.

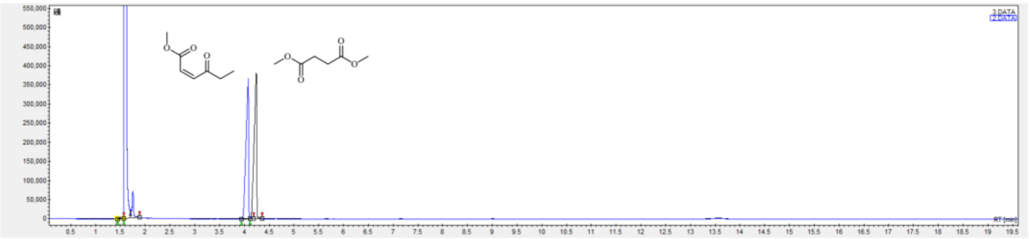

Figure S6. Representative GC chromatogram of **2**. Tr(**2**) = 4.07 min, tr(product) = 4.24 min.

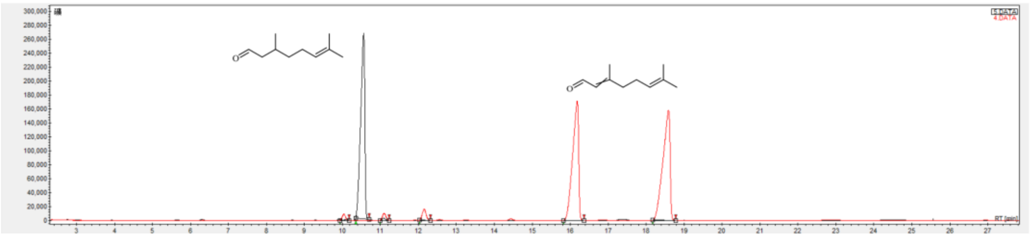

Figure S7. Representative GC chromatogram of **3**. Tr(**3**) = 16.18 min and 18.59 min, tr(product) = 10.56 min.

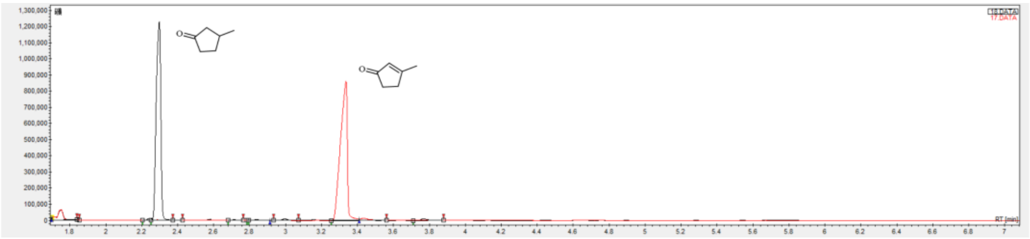

Figure S8. Representative GC chromatogram of **4**. Tr(**4**) = 3.34 min, tr(product) = 2.3 min;

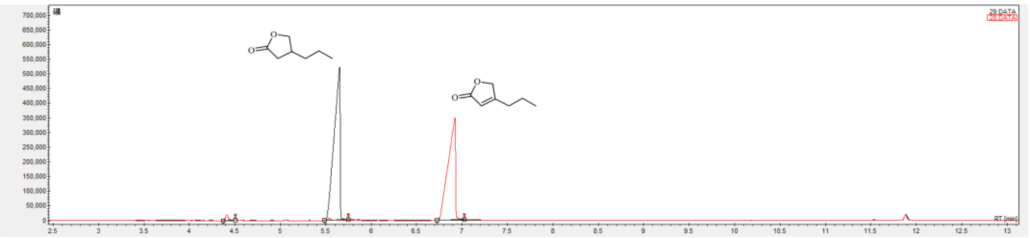

Figure S9. Representative GC chromatogram of **5**. Tr(**5**) = 6.92 min, tr(product) = 5.65 min.

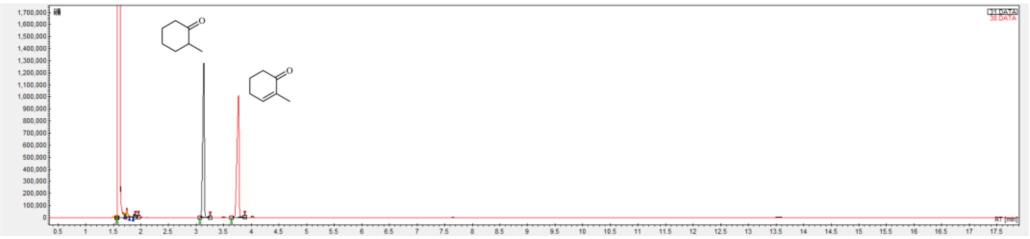

Figure S10. Representative GC chromatogram of **6**. Tr(**6**) = 3.77 min, tr(product) = 3.14 min;

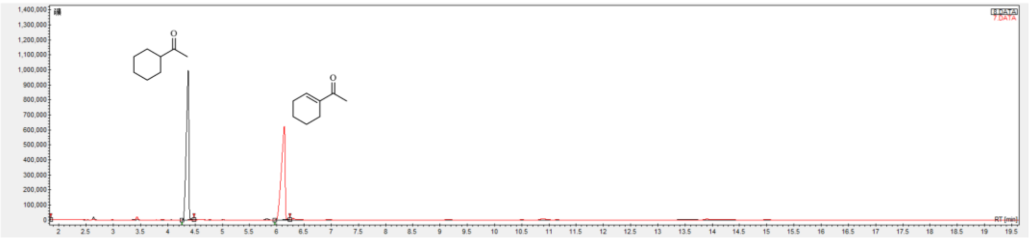

Figure S11. Representative GC chromatogram of **7**. Tr(**7**) = 6.14 min, tr(product) = 4.38 min.

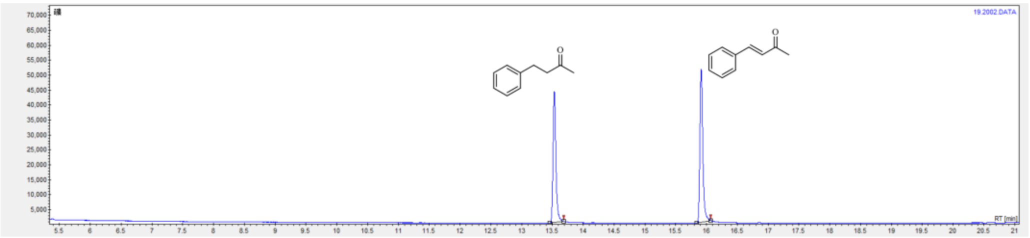

Figure S12. Representative GC chromatogram of **8**. Tr(**8**) = 15.97 min, tr(product) = 13.53 min.

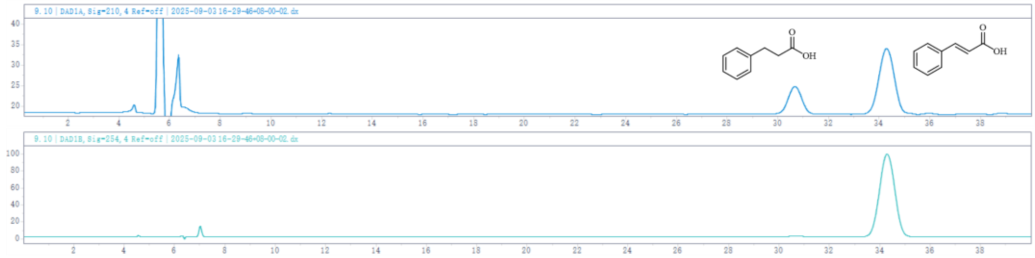

Figure S13. Representative HPLC chromatogram of **9**. Tr(**9**) = 34.28 min, tr(product) = 30.66 min.

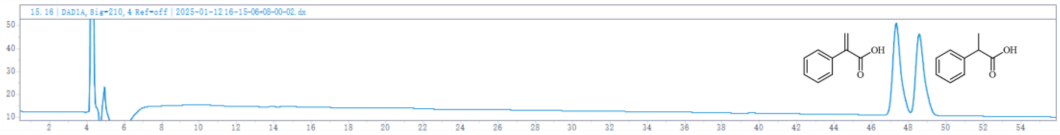

Figure S14. Representative HPLC chromatogram of **10**. Tr(**10**) = 47.30 min, tr(product) = 48.52 min.

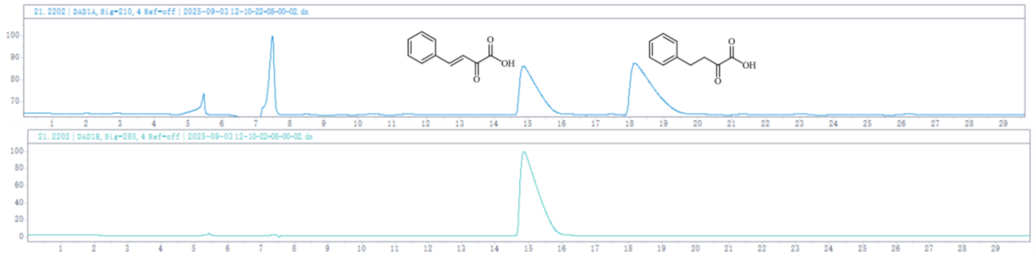

Figure S15. Representative HPLC chromatogram of **11**. Tr(**11**) = 14.86 min, tr(product) = 18.14 min.

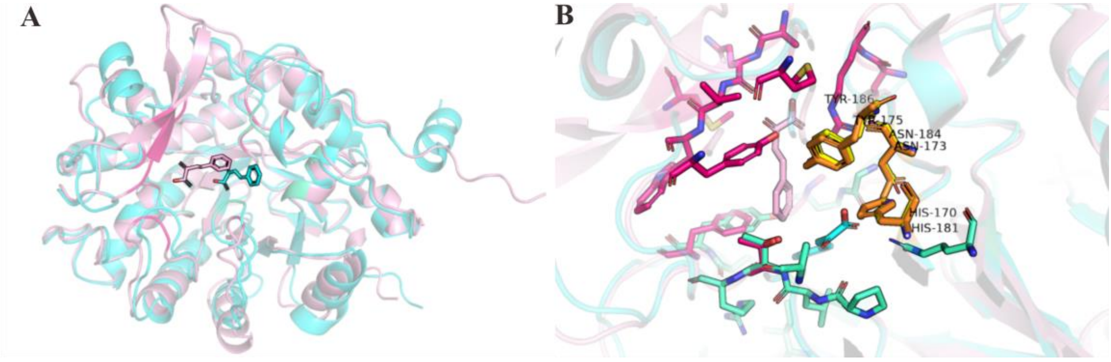

Figure S16. (A) Structural superposition diagram of S1gene1252928 and S2gene22028, (B) the distribution of catalytic residues within 5 Å of their respective catalytic sites. Orange-yellow-highlighted residues denote their predicted substrate-binding and catalytic residues.

4 Amino acid sequences of the mined ERs

The original gene sequences of the 41 ERs used in this study were submitted to the NCBI GenBank with accession Nos. PX562690-PX562730.

>S1gene1295291

YGLLSGIPFMRPMPEKVIEQTCDDYAISAKRAVEAGFDLLELHMGHGYLFSQF
MSPAYNTRRDKFGGSLENRMRFPRLALRRVREAVGPGVPIVVKLNLQDGFEG
GSTLDDCIEASKIIEQDGDASLLVLTGGFSAKSPMYLFRGPSAIKPLIAIQKNLIA
KLVYTLAAKKFPDMPFKEMFFLDDAKKVRAAVNMPIALVGGIKSMANFKTV
MNEDFDGIVLGRTLIHEPNLPKIYESGEKSTSGCISCNRC

>S1gene1417756

MTDSVLFSSFDWAENTLQNRMVLAPMTRGRAGADRIPNKIMGDHYVQRAD
AGLIITEATAISEEGIGWVDTPGIYTDKMVEGWRSVVNRVHEAGGKIVLQLWH
TGRASHSDFHDGNLPLSASAIKIEGDEIHTPKGKKPYEVPKAMTFDDIKRTVQ
DYKKAAVNAKAAGFDGVEVHAANGYLINQFLDSRSNQRTDNYGGSVDNRY
RFLAEVMDAVLSVWDKEHVGVRLSPNGVFNDMGADDFRETFTFVAQQLNKL
EIGYLHVMDGLAFGFHERGEAMTLAEFRALFDGMIIGNCGYTKEEAEKRLSD
GDGDMIAFGRPWITNPDLPTRFKHNYPLESFDDPSSWYGGGVEGYNDFETYR
EKSGKDAMTSLT

>S1gene278639

LFVNAAERCFDAGFDAVEIHMGHGYLLNQFLSPLDNNRTDEYGGNAENRARF
PARVLKAVKAAVGDRMAVIVKINVADGRSKGAQLEDGIVTAKVLEAAGADM
LVLSGGRNVESGWFTFGSNMNLEAMLRVIGKWSLSGMAIASTQAAVPKIEFK
ELYFWEYSTKIREAVKLPLAYLGGVKSADNVAQCMDAGFEAVQLARALLREP
DLVNRWRDEGGESLCDNCNSCVAYIYHPAGTWCIHR

>S1gene314101

MEALFKPIKINNLVVPNRIAMAPMTRSMSPNGVPTDQNLEYYKRRAAAEVG

MIITEGVEVSHPASSGYPNVPNLRQDSHEGWKKLISSVQEEGSTIFCQLWHVG
GIRKPGQPPNPEVPGYTPSGLVRKDKKVAYEMTDNDVEDLIQNYVKDIETIKM
LGFDGVELHGAHGYMIDQFFWRDTNVRKDKYGKEEDLRTKFVCEIVSRARK
LVGNDFPIMLRFSQWKQQDYEARLAPSPTDLETFLGPISDSGVDVFHASTRRY
WEPEFEGSDLNLAGWTKKITGKPTITVGSVGLDSDFIGLYVGNDKANTSSIKT
LVEMFDREEFDMVAVGRALLSDPEWVLKVKEGREEEIVGFTKEYVEKYF

>S1gene340638

GMEPDPNIPGYTASGIVRPGKKVAHAMTIDDIRETIDAYASDARICEEIGFDGV
EIHGAHGYLIDNFFWNGTNIRNDDYGGSIKNRSRLAAEIIKAIRSKVSSEFIVGL
RFSQWKQQDYAAKLAHNPDELKDVIKSPVEEGLDFLHSSMRRFWESEFKESD
ENLAYWTKKISGLPTISVGSVGLDSEFIDMSASASPTNINKVIDDINKEKYDLV
AVGRALLADHEWVLKIKDQRLDEINPYTKEALYKLY

>S1gene614360

MTILKEKLTLPCGAQIKNRICKAAMTERIAFSDNYTNQRHLNLYKKWAEGDIG
ILLTGNVQVDKKHLEGPANVCIEEETYIEQLPLLRKWAEEGTKDNTHLWMQIS
HAGRQTPGEINSSPKAPSSVQLKIPGRNYGIPSALTTEEIQEIIKKFTFVAKIARE
TGFTGIQIHSAHGYLLSEFLSPDINLREDEWGGTVENRSRIHVEIIKNIRREIGED
FPISVKMNSADFQKGGFTPDDSIQVAKIIEAAGVDNIEISGGTYEQPRLLGLDN
VSINPDRSEVRKESTIAREAYFLEYAEKIKKNIQIPLMVTGGFRTKQGMESAVK
SGACEIVGVGRPLCANPYAIKEMFDGKIEQLPIYEKTLSLGPWILSPSSPFRLIQ
ALNAFGAQAWFYQQIKRMGNNKLPDLNLGLFSAFRKDASEDKKAFKDLN

>S1gene633411

MLFEPFKLKNCEMRNRIVMAPMTRNQSPGGIPTEEVAKYYERRAKGEVGLIIT
EGIELSHKSSSAYPNVPRLDSEEARDGWKKVISGIKENNGSVIAQLWQCGGFR
KLGMGPDPDVPGYTASGLVKPGKKVAHEMTADDVKEAIEAYASDAEICENLG
FDGVEIHGAHGYLIDNFFWEGTNIREDIYGGSIEKRSNFVKEIVEAIRSRVTNNF
IVGLRFSQWKQHDFDAKLAQTPDELEKVIMPPVNAGLDYLHSSMRRFWESEF
PDSKENLAYWTKKISDITTIGVGSVGLDSDFIDMMAPANPTSIDKAIEDISSNK
YELIAVGRALISDPNWVIKMKEGRFDELIPYTKESLLTLY

>S1gene663605

MTNKSLFSPFSINNHELKNRFVMAPMTRNFSPEGIPSEYAPEYYAKRARGGVA
LILTEGVEVSHPSSSGYPDVPNLTSSESKKMWARVVEEVHKHDSKIFCQLWHV
GGIRKPGIDKNKDVPGYTPSGLVRANKKVAYEMTLDDIQEMISIYAEDAKICEE
LGFDGVEIHGAHGYLIDQFFWSDTNVRSDKYGGSLEKRTQFARDIIQACQNVT
TEDFSVGIRFSQWKQQDYDAKLALNENELKIFVDCLSESKPDFFHTSMRRFW
EPEMNNSSLAALVKGITDIPVIGVGSVGL

>S1gene1032130

MTKINDPLTLPCGQVIKNRVCKAAMTERIALGNNFTNEKHIELYKMWGAGDI
GILLTGNVQVHRDNLEGPANVAIEEDSYKEQMPMLKKWAEAATQEGSRLWM
QISHAGRQTPGEINMSPMSPSDVQLKIPGRKYGKPIPMTEEDIQDVINRFVFTA
KIARESGFDGIQLHSAHGYLLSQFLSPDINQRTDAWGGSLENRARIHLEIIKKC
REEVGNDFAISIKMNSADFQKGGFSPEDSIQVAKLFSDSGIDNIEISGGTYEQPR
LLGLDKVSINPKRSENRKESTIAREAYFLSYAEEIAKVVNIPLMVTGGFRTKEG
MEAALRDGACEIVGVGRPLCANPYAIKELLSGQIDELPKYEKTLSIGPWWLSP
TSPFRLIQAINAFSAQAWFYQQIKKMGKGLMPDLNLKPWKAFREDAKADQE
AIKEYQNF

>S1gene1055105

MQALFKPIKINNLVVPNRIAMAPMTRSMSPNGVPTDKNLEYYKRRAAAEVG
MIITEGVEVSHPASSGYPNVPNLRKDSHQGWKNLISAVQEQGSTIFCQLWHVG
GIRKPGQPPNPEVPGYTPSGLVKKDKKVAYEMTDNDVEDLIQNYVSDIETIKM
LGFDGVELHGAHGYMIDQFFWRDTNIRKDKYGKEEDLRTRFVCEIVSRARKL
VGNDFPIMLRFSQWKQQDYEALLAPSPEDLETFLGPISDSGVD

>S1gene115899

MEENMSSMGNIPGESLYSLYRYWAHGNLGMVITGNVMVDKGAMTGPGGVA
LEKDTDIAPFQKWAKIIKSNGALAIMQINHPGRQVFKAMKGKAIAPSAVPLDM

GKHSKLFAQPREMTCQDIHDVCKRFVQTAKQAEKAGFDGVEIHAAHGYLLT
QFLSPLTNQRQDEWGGSIINRARLLINIVSQVRAVCAKDFIVMVKLNSADFQK
NGFSFDDACEVVNRLEALGVDVVELSGGSYEAPAMQGQTRDDTTLAREAYF
LEFAQALVSKTDIPLMTTGGIKRAEVAEEVIEQGCALVGLASALAITPDLAKK
WQQEWAYSGIIPHCSWKDKSLASLANMAMVRRQLRRLGNNLTTLRNPSPLW
SLILDMLHRKKMTKRDVG

>S1gene430664

INEIIDGYRLSARYSREGGLDGVEVHGMSHGYLLNQFLSPATNFRKDNFGGSL
VNRMRIVTEILEATRAEIGPEMIMGMRINSDDGHEGGLSPDEWADIAKEFEET
GLLDYISCSHGTYINRMLIYPTAPEKHGFQLDATAQIKSKLKLPVVGVGRITTP
EEAETWLSQGKCDFVGMARALVADPKWAQKSLTGKSGTIRPCVGANWCMSR
IFAQAPLGCIHNPAAGQELDLDEYNLPPMKKKKRVAVVGGGPAGMRASWTLA
RRGHEVTLFEARAELGGQVRWWAQAESRHE

>S1gene2918962

MTENPFTILGSPIKLGPKTAKNRIWMTAHATLLVKDHLFTDAHIAYYAERARG
GAAVITMEAMATHPTTQPYKGKAFAFDPRMVPEYQKLADAVHVYGTLLLSQ
PWHRGRQTNGVTNGLPVWAPSAVPCAVYREMPHVMTTEDINEIIDGYRLSAR
YSREGGLDGVEVHGMSHGYLLNQFLSPATNFRKDNFGGSLVNRMRIVTEILE
ATRAEIGPEMIMGMRINSDDGHEGGLSPDEWADIAKEFEETGLIDYISCSHGTY
INRMLIYPTAPEKHGFQLDATAQIKSKLKLPVVGVGRITTPEEAETWLSQGKC
DFVGMARALVADPKWAQKSLTGKSGTIRPCVGANWCMSRIFAQAPIGCIHNP
AAGQELDLDEYNLPPVEKKKKVAVVGGGPAGMRA

>S1gene3357892

MEQLFKELTFSNGVKAKNKFLLAPLTNMQSPNQGRMSDDEFLWLTKRAEGGF
GIIMTCAMPVLKSGIGWKGQLGIYDPRHEDGHYRLNERLHNLEALSIAQIFHA
GIRADINYSGDKIGPSMNSEKSAREMSIAEIEEMKAAFVECAIRAQQCNYDGIE
LHAAHGYLIGQFLSKKFNKRGDTYGGSFKNRAKFLIDIIKEIKEKTSSNFLIGV
RISPERFGLDTNEMITLYKTLCENNNIHFIDISLWDSFKLVDDGPFTGESLLKLF
TTIDRGSKLLTVAGKIFSYEDIEILQNNNVDFFCLGRAAILDHQFPNRLKAEGS

NFQPYSAPVTRDHLLKEGLSETFVNYMATWKNFVSDSA

>S1gene2630108

QSFARGGAGIVIVEATAVSPEGRITPNCLGIWKDEHITGLKNIAERIKSQGAVAG
IQIAHAGRKASANRPWEGDDHIQPTDEKGWQPIGPSAQAFGAHLPVTPTEMT
KADIERVKNDFVKGAQRALEAGFEWLELHFAHGYLAQSFFSKHANKRTDEY
GGDAQGRGRFLLETLSAVRAVWPENLPLTVRFGVIEYDGDDEQTLSESIELIKQ
FKEIGADFVSVSVGFNIPDANIPWGPAFLAPIAKRVRDEADIPVATAWGVDTPE
LANDTVESQQLDVVMVGRMHLTNPHWTYFAAKKLGVEKPSWVMPAPYAH
WLERYAPSDER

>S1gene941552

MEVFFKPITVNKLTIPNRFAMAPMTRSRSPDGVPGENVRDYYQRRAAGEVGLI
ITEGVEVSHPSSSGYPDVPGFRDKADEGWKQVIDAVHSEGSKIFPQLWHVGAF
RKPGMAPDPEVPGYTPSGLVNANKKAAHEMTAEDVEMLIDCFVGDIVKIKDL
GFDGVELHGAHGYLIDNFFWEGTNQRADGFGGSIDKRTAFACEIVKRARAKV
GEDFPIAIRFSQWKQQDFEAKLAYTEEDLKTFLMPLVDAGIDMFHASNRRYW
EPEFEGSNLNLAGWTKKLTGIPTITVGSVGLKSDFIGLYAGDDAVESQSIDDLIK
RMESDEFDMVAIGRALLSDPEWVKKVKTGDFEAIIPFEKKYASEVYF

>S1gene1579967

METLFNPIKINNLHIPNRFAMAPMTRNKSPNGIPGQNVADYYERRAKGGVGLI
ITEGVEVSHPSSSGYPDVPGLKPEAHEGWRNVISQVHKYDSKIFCQLWHVGAI
RKPGLPPKPDVPGFTPSGLVRKDKKVAYEMTAEDVENMIEIYVRDIEIIKDLGF
DGVELHGAHGYLIDQFFWEDTNQRKDGFGGSIQLRSSFASEIIKRARAKVGND
FCIAIRFSQWKQQDYEAKLAKNQDDLETLLRPLVDAGVDVIHASNRRFWEPE
FEGSNLNLAGWTKKITGLPTITVGSVGLESDFIGFYQGEDEVKSAPIDDLVKR
MDDKEFDMVAVGRALLSDPEWVNKVKEGRYDEVIPFSKKHAENYY

>S1gene2373022

YPDVPNLTSSESKKMWARVVEEVHKCGSKIFCQLWHVGGIRKPGIDKNKDVP
GYTPSGLVRANKKVAYEMTLEDIQEMISIYSEDAKICEDLGFDGVEIHGAHGY
LIDQFFWSDTNVRSDKYGGSLEKRTQFARDIIQACQNVTTEDFSVGIRFSQWK
QQDYDAKLALNKNELKIFVDCLSESKPDFFHTSMRRFWEPEMNNSSLAALVK
GMTDIPVIGVGSVGLDKDFIRLYAGDDKTKISDFDQLFDSFQAEEFDLIAIGRA
LLSDPDWVKKLEDNETDTIVPFDKSFVENYV

>S1gene435640

MLFEPYHLKGLSLRNRIVMAPMTRNQSPGGIPTKEVIEYYKRRAKAEVGLIIT
EGIEISHKASSAYPNVPRLDTKEAIDGWKKVVSGIKENNGAVIAQLWHCGGFR
KLGMDPDPLVPGHTASGIVRPGKKVAHAMTVDDIKETIEAYASDAKICEEIGFD
GVEIHGAHGYLIDNFFWSGTNKRNDSYGGSIKNRSKLATDIVKAIRSNVSNQFI
VGLRFSQWKQQDYEAKLANSPNELKELINDPIEEGLDFLHSSMRRFWESEFEG
SDENLAYWTKKLSGLPTISVGSVGLDSDFIDMSAPASPTNINKAIDDINQKKYD
LVAVGRALLADHEWVVKIKEGRVDDIVPYSKEALLKLY

>S1gene770360

MIFEPYKLKNITLRNRVVMAPMTRNQSPGGIPTEGVVSYYSRRSKAEVGLIITE
GIEVSHKASSAYPNVPRLDSKNAREGWKKVVNGIKKHDGSVIAQLWHCGGF
RKLGMQPDPEVPGYTASGLVKPGKKVAHEMTLEDIKETIEAYASDAKICEELG
FDGIEIHGAHGYLIDNFFWSGTNIRDDRYGGSLEKRSQFVVDIIKAIRDQVSNE
FIVGLRFSQWKQHDFEAKLALNPEELEKVLIEPVNSGLDYVHSSMRRFWEKE
FENSEENLAYWTKKITKIPTIGVGSVGLDSDFIDMTAPASPTSIERAIEDISKNK
YDMIAVGRALLSDPEWVLKMKEGRLNDVIPYTKEALLNLY

>S1gene2129020

MIFENFQLKNITLRNRIVMAPMTRNQSPGGIPTQEVVAYYSRRAKAEVGLIITE
GIEVSHKASSAYPNVPRLDSNEAKEAWKKVVEEIKNNNGAVIAQLWHCGGFR
KLGMQPNPEVPGHTASGLVKPGKKVAHEMTLNDIKETIDAYASDAKICEEIGF
DGVEIHGAHGYLIDNFFWSGTNVREDEYGGSIENRSQFVSDIIKSVRENVSENF
IVGLRFSQWKQHDFEAKLASNPEELKTILTSPVESGLDYLHSSMRRFWESEFE
GSKENLAYWTKKITKIPTIGVGSVGLDSDFIDMTAPATPTSIDKAIDDITNDKYD

LIAVGRALLSDHEWVLKMKEGRVNDVIPYTKDALLNLY

>S1gene2614772

MATNALFKPYSHGNLSLTNRIVMAPMTRQFSPNGIPTPDVAAYYKRRAQGGT
GLIITEGTTVNDNVATMDANIPQFHGEQALSGWQTVVNEVHSVGGKIMPQLW
HVGMARVAEKAPFPDLPSAGPSGLFKPGKQGAEPMTVQHIESVIAAFAQAAA
DAKSIGMDGIEIHGAHGYLIDQFFWEGTNQRSDSWGGSMENRGRFAVEIIKAI
RAATGPDFPIILRYSQWKQQDYTARLAHSPQELEQFLLPLSEAGVDVFHCSQR
RYWENEFEGSNLNLAGWTKKLTGKPTITVGSVGLNDDFFGAFKGQDSSTRSV
DDLLERLDAGEFDLVAVGRALLQDPNWANKIKENRTDELEQYSGKALATLS

>S1gene1252928

MSETLFKPLELGSLNLPNRIALAPMTRARTTQPGDIPNAMMAEYYAQRSGAG
LIISEATQISSQGKGYSFTPGIFTQEQIEGWKLVTDAVHKSGGRIFLQLWHVGR
MSHESLHADGKPVAPSALSPDAQVWIVDPETGEGGMVDCPVPRALSAAEIKE
VIQDFRQGAANAIAAGFDGVEIHGANGYLIDEFLRRSSNQRTDAYGGSQDNRI
RFLREVTEAVAGEIGADRTGIRLSPYITQRNMADDEIIEVILKAAGALNDIGIAYI
HLSEADWDDAPIVPEQFRHELRKAYSGAIIVAGKYTQERGEKIIGDGLADIVAY
GRPFIANPDLPRRFAEGRPLAEFDPGTLFGGTEAGYSDYPVAPPASKTA

>S1gene19368

MKLFETLSLAGKTLNNRVVMPPMTRSRSTQPGDVPNTLMAEYYAQRASAGLI
VSEGTQISALGKGYAWTPGIYTKAQIDGWKLVTDKVHEAGGVMFAQLWHVG
RVSHPSNTEGQQPISASAIQAKGVKVFVDEGGNPGFVESVMPREMSIEDIKAV
VEEYRVAARNAVDAGFDGIELHSANGYLLNQFIDSQSNTRTDEYGGSVQNRIR
FLKEVVQAVSEEIGADKVGVRLAPLTTLNGTVDDNPEETYLAIASMLNSFKIG
YMHIAEADWDDAPQMPISFKMAIREAYSGLLIYAGKYDTERAEQALTEGWAD
MIGFGRPFVANPDLPYRLANNVPLNEHNPDTLFGGGEKGYTDYSFAK

>S2gene1134

MPIPKLFQPIKVGNVELKHRVVLCPLTRYRATSKHVPTDLQAQYYAQRASTPG
TLLITEATAITERAGGYNNIPALETGEQVEGWKKVVDAVHEKGSYIFAQLWAL
GRTANPDYMAKKGYDIVSASDVPLKDKPKPRPLTIPEIKEYLIWYAQAATNAV
KAGFDGIEIHAANGYLPHQFLDDTTNLRTDEYGGSVENRARFTLEIVESITKAI
GPTKTAIRLSPFETFQGHGTYFEPQSLDFMRNIWAPRPFISAGFFTPELALEVAE
TKGDLTAFGRSFLANPDLPYRIKNNIPLNQPDYSTFYTPENPVGYIDYPFAEKK
EAL

>S2gene1137

MSTPKLFQPLKVGNVQLKHRIVLSPMTRYRATSKHIPSDLQVQYYGQRASTP
GTLLITEATAITEKAGGYNNIPALETDEQVGGWKKVVDAVHAKGSYIFAQLWA
IGRTADPEYMAKRGYDLVSASDVPLKDRPKPRPLTVLEIKEYLAWYAQAATN
AIKAGFDGVEIHAANGYLPHQFLDDTTNLRTDDYGGSIENRARFTLEIVESITK
AIGSSQTAIRLSPFETYQEMYMKDTIPTFTYVANKLAEKFPDLAYVHCIDPRTK
EGDQEGKSLDFMRKIWAPRPFISANYYTPELAREVAETKGDLIAFGRSFLANP
DLPYRIKNNLPLNQPDYSTLYTPENPVGYIDYPFSEKKEDTL

>S2gene1139

MSTPKLFQPIKVGEVELKHRVVLAPLTRFRASIKHVPSDLQAEYYAQRASTPG
TLLITEATFITEKAGGYNNVPALETEEQIAAWKKVVDAVHAKGSYIFAQMWAL
GRAANPEYLNQKGYDLVSASDVPLNGKPKPRPLTVEEIKEYVGWYRQAAINA
VQKAGFDGVEIHSANGYLLHQFLEDHSNLRTDEYGGSIENRARFTLEVVDAV
TKAVGPSKTALRLSPFETFQQMGMADPYPTYTYVVNQLNEKYPNLAYLHCV
DPRSKGIVQLETDLQGKSLDFIRKIWQPRPFVSAGLYTPELALDVAEQKGDLIA
FGRFFISNPDLPYRIKNKIPLTPYNSSTFYVPESPVGYIDYPFAEKKEETTTPQST
L

>S2gene8059

MAPPIINAPAPGVPFFTPGQFPASGTAVNPQPENKSIPTLFQPLKIRGLEFHNRIW
LSPLCQYSAENGIVSPWHLAHLGGIISRGPGLSFIEATAVSPEGRITPEDVGIWS
DAHIAPLRQIVDFAHSQNQKIAIQLAHAGRKASTVAPWLDSGAAASKEAGGW
PDDVVGPSNIPFSDRFPKVKALTKEGIQRIVQAFVEGAKRALKAGFDVIEIHNA

HGYLLSEFLSPTVNDRTDEYGGSFENRARLTLEVVDAIRAAIPPEMPLFLRVSA
TEWLEQVLPDVPSWRVEDTVKLAGLLADHGVDLLDVSSGGINSQQKIIAKGA
YQAVFSEAVKKAHGDKILVSAVGNITNGHIAQEILDKGQADAIFVGRYFQKNP
GLVWSFAEDLGVSITVAHQIEWAFAGRRHGVGRTHVKAAKGTTKL

>S2gene1898

MTRAMSKLFVPLDVGTVQLQNRCVLAPLTRYRCDDDWVPLPMTKEYYTQR
AAAPGTLLISEATFISEQAAGRRNVPGIWTDAQVAGWKPITDAVHARGCFMFC
QIWHLGRAGWPDVHRSLGHRVLSSSAVAIDATRPVPEAMTEDEIWAVISDYAS
AAKRAMAAGFDGVEIHGANGYLADQFLQDKCNQRQDAWGGSIENRARFHL
EVTKAVVEAIGANRTAVRLSPFSDYLGMLMDDPLPTFEYLVRQLRPFKLAYLH
LIEARITGNDDGDCGGSNDCSSMIKSWNNQSPIVLAGGFQADSARRAVDETY
KDYDIAIGFGRYYISNPDLVFRIREGIELVKYNRSHFYTPKLAEGYIDYPYSAQ
YLAQTK

>S2gene8654

MVSSKLFEPLKIGNMELKHRVVMAPLTRYRADKQHVPLPLMKEYYSQRASTP
GTLLITEATFISQQAAGDANVPGIWSKDQIAGWKQVTDAVHAKGSYIYVQLW
ALGRVAKPDVAKAEGFTIKAPSSIPTDGLPAPAELTAADIESFVADYAQAARNAI
EAGFDGVELHGANGYLVDQFIQDVSNQRTDDYGGSIENRSRFPLRVLEALVAA
VGSSRVGLRLSPWSDFQGMRMADPVPQFTHLVSEVRKLKLAYLHIVEARVSG
NADTESANTNNFIFKAWQKASPVLVAGGLKPDTARALLEKDHDEVEVAAVFG
RYFLANPDLPFRIQKGLELNQYNRHTFYDAESPVGYIDYPFSQEWLSVQA

>S2gene25897

MESTKLFQPLSLGKLELKHRVIMAPLTRLRCDRDHVILPIAKEYYAQRASSPGT
LIIAEATMPSLKHGFGDHTPGIWSDAQIKSWKEITDTAHDKGCFMYLQICLPG
RAAAEGYECLSSSAVPIDAQSRVPKEMNEEEIQEAIESVVQASKNALVAGFDG
VELHGANGYLIDQFLQDNCNQRTDKWGGSVENRSRFALELVAAVAEAIGSDR
LGLRLSPWSEFQSMRMKEPVPQFSHLVRELKKYKLAYLHLIESRVNNWADKE
KTEGIEFLIDIWGNQSPVLVAGGFKLDSAKNAVDSEYAKYDTAVVFGRYYLST
PDLVFRLQHGLEPNQYDRNTFYTPVQPEGYLDYPFSKEFEKGAKSQA

>S2gene28498

MAENTKLFTPLQVGSTQLEHRVVMAPLTRFRATREHVPTDVMARYYAQRAV
VPGTLILSEATFITAKAGGYSHIPGLWSEEQLAAWKKVTDGVHARGSKMYVQ
MWHLGRAAWPDPVASGGAVRDEDDFDFEHDFVSSSDVVMADGLPAPRPLTE
EEIWSTIADYATAARNAVEKAGFDGVEIHAAHGYLIDQFTQDTANRREDAWG
GSIENRSRFAVEVAKAVAAAVGADKVGIRLSPWSTWQGMRMADPVPQFSHLI
KQLSQLGLSYLHIVEPRVHGVMDTDPKNESLDFALENWGREKPIFVAGGFTTE
LAKQAVEEKYRDHPVAVVFGRRFISNPDLVYRVKKGLPFNDYDRMTFYINQR
LGHKVEPGYIDYPYSEEYIKEFGKPDVAV

>S2gene28781

MSKLLTPLRLGRVALGHRIALAPMTRMRADDKHTPLPSVKEYYGQRASVPGT
LLITEGTIISPEHNGYPNLPEIYTESQIAAWKEVTDAVHEKGSFIFLQIAALGRA
ANPGLIAQQGYQLVSSSDVPMKSPFSNEVHYPVALTEQGIQDTIAAFAKAAQN
AIAAGFDGVEIHGANGYLVDQFIQDVSNTRNDGWGGSIEKRSRFALEVTRAVA
NAVGSDRTAIRLSPWSKYQDMGMADPLPQFTYLAQELAKLKLAYIHACESDT
RNGPSVSFLLEAYGNASPVVVAGGYDSRTAKQAVDVDYKDHDVVVAFGKPFI
SNPDLPHRIAKDIAFEASDPSTHYGQSSEGYIDYKFSADFVAVKA

>S2gene6523

MPKADSPSPQYVPLKDTKLFSPLKLGAVELSHRIIQAPLTRMRAEKESHGVHV
PGSRVVEYYSQRATKGGLQLTEATDICLDASAYPGCPGIFTESQIAGWKAVTD
AVHAKGGFIFNQIWHTGRASGPGMLNSKVSLSSTSQPMKGKYLDGSDCAENP
PKPMTVEEIHATTAEFAAAAKRAVSAGFDGVEIHSANGYLLEQFLHDNVNTRT
DEYGGSVENRCRFTLEVIKAVTDAVGADRVGIRLSPFNYFQDTKDSDPNAHW
LYLCEQIAALKESQRPCYVHMVEPRFDEVLDEEQKLAELSSYTSSETGSTKKK
NSLTPFRKALQPAGIKFLAAGSFTRDNAGPKVEEDLADGIVMGRFFIANPDLV
ERLKEGLSLNAYDRTTFYGASPPEKGYLDYPFYEATKAVEVQA

>S2gene4448

MASQTFENVAASGISYFTPAQEPASGTCLDDESTAKLFTPLQIRGVTLPNRLILP
PLCQYSAKDGYATDWHLTHIGGIIQRGAGLTIMEATGVLPRGRITPQCLGLWE
DGQIEPLRRITEFAHSQNQKIGIQLAHAGRKASGVAPWLSYVATSTEEVGGWP
NDLHAPSAIAQDDNAPIPKAMTLEDIEDLKTAFADAAKRAVTAGFDVVEIHSA
HGYLLHSFLSPVSNKRTDRYGGSFENRTRVLLEIVTGVRDVIPETMPPLVRISG
TDWFEFDEALRAEFPDTWTVEQSGRLALLLAERGVDLLDVSSGGVHAKSVA
AIRPGPAYQVPFSQEIKKVVGDKLLVSAVGGIKSGTLAEEVLQSGLDAVMCGR
WFQKNPGLVYAYADELGVEVKIANQIGWGFMGRGRKGKKTTA

>S2gene19529

MSSEAISNKGASGVSYFTPAQEPPSGSQLNGSTKLFSPLTIRGTTFQNRLFLPPL
CQYSAKNGYANDWHLTHLGGIIQRGPGLTIMEATAVQPIGRITPEDLGLWEDG
HIEPLRRITEFAHGQNQKIAIQLAHAGRKASCVAPWLSANAVATKDIGGWPDEI
VAPSAIAQEAVNPVPKALTLDDIEKLKQDWVEAAKRAVKAGFDVIEIHSAHGY
LLHSFLSPVSNKRTDKYGGSFENRTRLVLEIAEAVRAAIPKEMPLFVRISATDW
FEFDASFKEEFPETWTVAQSSELSSLLAERGVDLIDVSSGGVHAKSVAAIRPGP
AYQVPFAQDIKKAVGDKILVSAVGGIKTGQLAEEVLQSGIDVVMAGRWMQKN
PGLVYQFADELEVDVKMANQIGWGFGGRGNRGARKNRL

>S2gene28035

MTKSPLENTPAAGTPYFTPAQDPPAGTALHPESAPTLFQPLAIRGVTLQNRFVV
SPMCQYSAEDGHHTDWHFAHLSQFVLRGSALTIVEASAVMANGRITPEDSGL
WKDSQVAPLRRIVTFAHAQGQKMGIQLAHAGRKASTVAPWIAPRQGGSSATA
TPELGGWPDNVWAPSPGKFSDGFPEPREMTKDDIAAVVAAFGESAKRAVEAG
FDVIEIHGAHGYLINAFMSPLSNRRTDEYGGSFENRTRLLFEIVKAIRQVIPDA
MPLFLRISATEWMEYAGDPSWTLQDSIQLARLLPAAGVDLLDVSSGGNNAHQ
KIEINPYYQVNLAGAIRDAVKKDGQKQLLIGAVGLITDAEMARSIVQEQSDAE
TQAQRDRRPSCGTIEVEQEGTGERTQADLVLLARQLLREPEFPLRVAHHLGVE
VAWPNQYHRAGWRKSQRV

>S2gene11210

MTEHIEKPLTLASGLTLQNRLVNAAMAENMADKNGLPHQKFRTPYSVWAKG

GWGMVLTGNVEVDRRYLGAPGDIAFNDDIPYEEMLAAWRTWAEACNAGGT
PTLVQINHPGRQSPMGAGSRGLFAKNLAPSAVPLNFGSGLLPKLISTLMFGTPK
AMTQADIDDVVRRFAATAKLSADAGFAGAEIHAAHGYLLAQFLSEKTNQRTD
AYGGSPAARAKIVVEVIKAMREATPKGFTIGIKLNSADHQSSAELAACLEQLK
AITAASVDFIEISGGSYESPTMNTGPADSEEKANKSARTKAREAFFLEFAQAIR
KDVPDVPLMVTGGFRTRQGLEAALHDGGCDLVGIGRPACVKPLLPNEVILNK
AIKDDDAVFHVQKIQPPWIATKLGIKIIGAAGDSCTNLAVIQAHYQGQLQKIGQ

>S2gene14638

MLRYGFAAGLVALQFASQAVTGVHGSLGSRDSATESLQTFSYKPRIFILSDILNE
PDDSMSLVRYLLYSNEFNTTGLCATTSWWLQNETHPEEMERIINAYGQVVDN
LNQHVHPSVQYQSPDELLQLVTSGPNVYGSAALDEPISEGAQHLIAALQASED
PLFVTAWGGTNTLAQALQYMDENLSESEASALRSRIRLYTISDQDDTGAWIRA
RYPEIFYIISVHAWNDYTLATWTGINLASCACVDNTTVLNPWLDANVRLGPLG
AVYPQIEYGMEGDTPSYLWLVQNGLVYRDRIDYGTWGGRYNLPQAPVDVAK
GRAHISNQYVNSLDAVIGADGESYSTHQATIWRWRSAYQDDLAARMQWTLT
PNFTDAGHPPVINVNGHEGPDPLFLTVAANQTYLLDAGLTVDPDAGSAGGNR
NLAFDWALYPEPTKFLSAYLTVPITAVNGTGEVLATNAAGFANATVGTAVQFT
APEVFTNPNTGVSMDFHLLLSVTNSAGKYPITRYLRIVCQYES

>S2gene22028

MSASRLFEPLKLGNVTLQHRIAMAPLTRFRADENHVPLPFVKEYYAQRASVP
GTLIISEATLISKKAGGYGLVPGIWSQEQIAGWKEVTDAVHAKGSSMFLQLWA
LGRVADPGNAAKEGIEIKAPSAIPQAEGATVPQELSRDEIKSFVADYAQAARNA
IAAGFDGVEVHGANGYLIDQFTQDTANQRADDYGGSVENRSRFAVEVATAVV
DAVGPSKVGMRLSPFSDFQGMHMADPLPQFTDLTRKLKALKLAYLHVVESRV
AGNADQESTDKVDFIVDEWDNSSPVLIAGGFTPASAKRAVDEEYKGKDVIIVF
GRHFISTPDLPFRVQKGIELTPYDRDLFYNAGEKKGYTTYPFSEEFQSQQGAKI

>S2gene13870

MADTHYGSAGVDPSPLGQPLEFPVSKRVAINRFLNGAMSEGLATYHETDLSA
RGIPGKDLAQLYGKWGEGKWGQLLTGNVMIDPGHLEAPGNMIVPVDAPFEG

ERFEGFKAIATAGKAHGSLIVPQVSHPGRQVPDNVQKHPISASDMQLITPAMG
RTYAKPRAATKEDIEGVIKGFTHAAVYLEKAGFDGIQLHGAHGYLLAQFLSPT
TNKRTDQYGGSLENRMRLILEIAASIKAQVSPSFVLGIKVNSVEFQEEGFTPEE
AILLCQALEKAGFDYVETSGGTYESNGFVHKKESTRKREAYFIEFSEHIAKSVS
SLKVYTTGGFKTVDGMVKALESVHGVGIGRAACQDPDLPELILAGKATGVG
KYSIEEDNIFIRLVAAKMQIQQIANGQKPDELTDPAVIQKIQASLGMPSQL
